# HIF1α-glycolysis engages activation-induced cell death to drive IFN-γ induction in hypoxic T cells

**DOI:** 10.21203/rs.3.rs-3830704/v1

**Published:** 2024-01-12

**Authors:** Hongxing Shen, Logan Mullen, Oluwagbemiga A. Ojo, Chuan Xing, Abdelrahman Yassin, Zach Lewis, James A. Bonner, Lewis Zhichang Shi

**Affiliations:** 1 Department of Radiation Oncology, Heersink School of Medicine, University of Alabama at Birmingham (UAB-SOM), Birmingham, AL 35233, USA.; 2 Genomics Core Laboratory, Institute of Arctic Biology, University of Alaska Fairbanks, Fairbanks, Alaska, 99775, USA.; 3 O’Neal Comprehensive Cancer Center, UAB-SOM, USA; 4 Department of Microbiology and Immunology Institute, UAB-SOM, USA; 5 Department of Pharmacology and Toxicology, UAB-SOM, USA; 6 Immunology Institute, UAB-SOM, USA

**Keywords:** T cell, HIF1α, anaerobic glycolysis, IFN-γ, hypoxia, ICB, acetate supplementation

## Abstract

The role of HIF1α-glycolysis in regulating IFN-γ induction in hypoxic T cells is unknown. Given that hypoxia is a common feature in a wide array of pathophysiological contexts such as tumor and that IFN-γ is instrumental for protective immunity, it is of great significance to gain a clear idea on this. Combining pharmacological and genetic gain-of-function and loss-of-function approaches, we find that HIF1α-glycolysis controls IFN-γ induction in both human and mouse T cells activated under hypoxia. Specific deletion of HIF1α in T cells (HIF1α^−/−^) and glycolytic inhibition significantly abrogate IFN-γ induction. Conversely, HIF1α stabilization in T cells by hypoxia and VHL deletion (VHL^−/−^) promotes IFN-γ production. Mechanistically, reduced IFN-γ production in hypoxic HIF1α^−/−^ T cells is due to attenuated activation-induced cell death but not proliferative defect. We further show that depletion of intracellular acetyl-CoA is a key metabolic underlying mechanism. Hypoxic HIF1α^−/−^ T cells are less able to kill tumor cells, and HIF1α^−/−^ tumor-bearing mice are not responsive to immune checkpoint blockade (ICB) therapy, indicating loss of HIF1α in T cells is a major mechanism of therapeutic resistance to ICBs. Importantly, acetate supplementation restores IFN-γ production in hypoxic HIF1α^−/−^ T cells and re-sensitizes HIF1α^−/−^ tumor-bearing mice to ICBs, providing an effective strategy to overcome ICB resistance. Taken together, our results highlight T cell HIF1α-anaerobic glycolysis as a principal mediator of IFN-γ induction and anti-tumor immunity. Considering that acetate supplementation (i.e., glycerol triacetate (GTA)) is approved to treat infants with Canavan disease, we envision a rapid translation of our findings, justifying further testing of GTA as a repurposed medicine for ICB resistance, a pressing unmet medical need.

## Introduction

Naïve T cells, upon receiving signals through T cell receptor (TCR) (signal 1), co-stimulatory receptor (e.g., CD28, signal 2), and various cytokines (signal 3), are activated and differentiate into distinct sub-lineages, including IFN-γ-producing T_H_1^1^, IL-17-producing T_H_17^2^, the immunosuppressive FoxP3^+^ regulatory T cells (T_reg_)^3^, as well as other recently-defined subsets^4^. Notwithstanding the essential role of these immune signals in deciding T cell fates, in recent years, accumulating evidence indicates that at the fundamental level, it is the cellular metabolism that orchestrates T cell activation and differentiation^5–15^, by meeting their drastically increased bioenergetic and biosynthetic demands^16^. Interestingly, seminal efforts from many groups reveal a selective but not universal role of metabolic processes and transcriptional factors in this process^4–7,10–15^. For example, we found that the initial metabolic reprogramming in activated T cells, switching from fatty acid/pyruvate oxidation via the TCA cycle to the glycolytic, pentose-phosphate, and glutaminolytic pathways, is primarily controlled by Myc but not HIF1α in T cells^9^, even though both are master regulators of cellular metabolism, downstream of mTOR^17^. On the other hand, HIF1α-glycolysis orchestrated a metabolic checkpoint in T_H_17 differentiation and induction of T_reg_ (iT_reg_)^8^. However, given the shared importance of glycolysis in both T_H_1 and T_H_17 differentiation, HIF1α is not required for IFN-γ induction/T_H_1 differentiation^8^. While this could be due in part to greater HIF1α induction in T_H_17 cells than T_H_1 cells^8^, it is noteworthy to mention that we performed those studies primarily under the regular normoxic culture condition commonly established in the laboratory (21% O_2_). It remains to be determined whether and how HIF1α controls IFN-γ induction in T cells, under hypoxia.

Hypoxia is a prominent feature in various physiological and pathological settings. Physiologically, only the tissues directly exposed to inhaled atmospheric air (such as the upper airways) have an O_2_ level at ~21%. Most other healthy tissues experience O_2_ deprivation (hypoxia), to a certain degree. For example, the O_2_ level in arterial blood is about ~14%^18^, and this is reduced to 5–6% in the interstitial space^19^. In lymphoid tissues like spleen, it is mostly ~3–4%^20^ but lower in the germinal center^21^. In the gastrointestinal (GI) tract that contains ~70–80% of the total lymphocytes in our body^22^, a wide range of O_2_ tensions exists, being almost anoxic in the lumen, where many obligate anaerobic commensal bacteria reside, and slightly increased at the base of the villi^23^. The intestinal tissue, including the lamina propria where many T cells are found, has an O_2_ level of ~7%^24^. Pathologically, extreme hypoxia arises in the tumor microenvironment (TME) of solid tumors (~1% O_2_), as a result of abnormal vasculature, heightened metabolic activities of tumor cells, and other factors^25^. Likewise, severe hypoxia can be found in the inflammatory sites, due to edema, vasculitis, vasoconstriction (limiting oxygen delivery), and recruitment of polymorphonuclear cells that consume high amounts of O_2_^26^. As its name tells, hypoxia-inducible factor (HIF)-1 α subunit (HIF1α) is widely regarded as the primary regulator of cellular adaptive responses to hypoxia^27^. When O_2_ tensions are low, hydroxylation of the prolyl residues of HIF1α by prolyl hydroxylase domain (PHD) enzymes is inhibited; subsequently, recognition and ubiquitination of HIF1α by the von Hippel-Lindau protein (VHL)^28,29^ is suppressed. Additionally, hypoxia promotes the interaction between the C-terminal transcriptional activation domain (C-TAD) of HIF1α and other transcriptional coactivators through inhibiting hydroxylation of an asparagine residue within the C-TAD of HIF1α^30^. These modifications converge on the inhibition of proteasome degradation of HIF1α, leading to stabilized HIF1α, which then translocate to the nucleus and mediate transcriptional and epigenetic programs. Given the quite ubiquitous distribution of hypoxia in our body and the essential role of IFN-γ in immunity against intracellular pathogens and tumors^31^, a clear understanding of the role of HIF1α-glycolysis axis in IFN-γ induction in hypoxic T cells is of utmost importance.

Here, we report that HIF1α-anaerobic glycolysis is essential for IFN-γ induction in T cells, under hypoxia. HIF1α deletion (HIF1α^−/−^) in mouse T cells, knockdown (HIF1α^KD^) in human T cells, as well as glycolytic inhibition with 2-DG markedly reduced IFN-γ production in hypoxic T cells. On the contrary, HIF1α stabilization in T cells exposed to hypoxia, deleted of VHL, or treated with PHDs inhibitor (DMOG), increased their production of IFN-γ. We identified impaired activation-induced cell death (AICD) but not proliferative defects in HIF1α^−/−^ T cells as the major cellular mechanism underlying reduced IFN-γ production. Molecularly and metabolically, this can be attributed to the depleted intracellular pool of acetyl-CoA ([acetyl-CoA]), as acetate supplementation replenished [acetyl-CoA], re-engaged AICD, and restored IFN-γ induction in hypoxic HIF1α^−/−^ T cells. As a result of reduced production of IFN-γ and other late effector cytokines (e.g., granzyme B and perforin), hypoxic HIF1α^−/−^ T cells were less able to kill tumor cells *in vitro*. *In vivo*, tumor-bearing HIF1α^−/−^ mice were resistant to combined anti-CTLA-4 and anti-PD-1 therapy (ICBs). Importantly, administration of acetate to tumor-bearing HIF1α^−/−^ mice overcame this ICB resistance and restored IFN-γ production in tumor-infiltrating T cells (TILs). Collectively, our results establish T cell-intrinsic HIF1α-glycolysis pathway as a major regulator of IFN-γ induction in hypoxic T cells. They manifest acetate supplementation as an effective therapeutic strategy to bypass ICB resistance associated with HIF1α loss in T cells. Considering acetate supplementation (GTA) is approved to treat infants with Canavan disease, our study lays a solid foundation for future clinical testing of GTA as a repurposed medicine for ICB resistance, a pressing unmet medical need in ICBs.

## Results

### HIF1α controls IFN-γ induction in hypoxic T cells.

**Figure 1.**
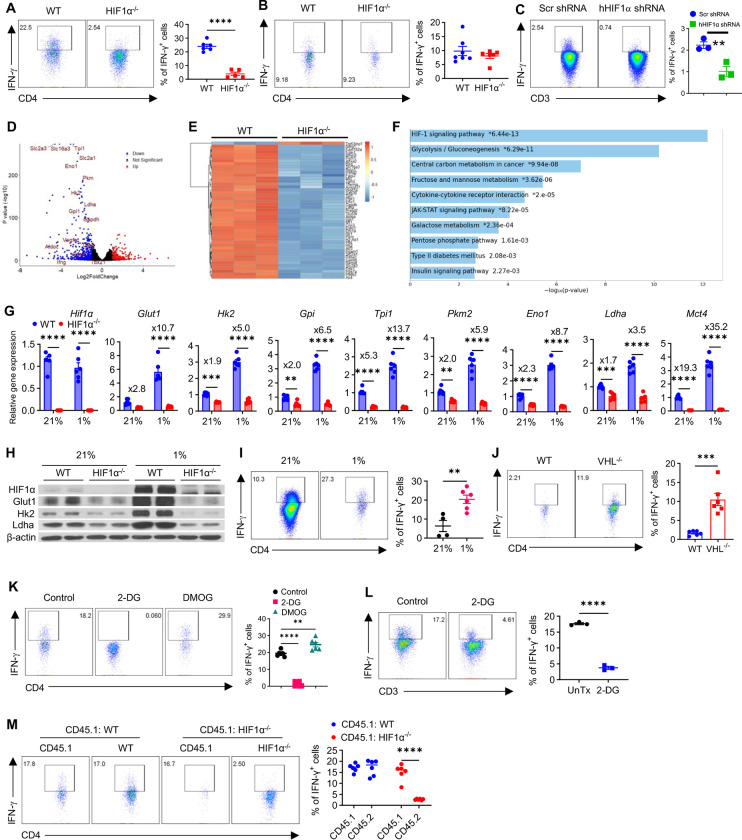
HIF1α-glycolysis controls IFN-γ induction in hypoxic T cells, *in vitro*. **A**-**B**. Naïve CD4+ T cells isolated from WT and HIF1α^−/−^ mice were activated under hypoxia (**A**) and normoxia (**B**) for 5.5 days, followed by detection of IFN-γ. **C**. Naive human CD4^+^ T cells isolated from PBMCs of healthy donors were activated, transduced with retroviruses expressing scrambled shRNAs (Scr shRNA) or shRNAs against human HIF1α (hHIF1α shRNA), and cultured under hypoxia for 5.5 days, followed by detection of IFN-γ. **D-F**. Total RNAs extracted from WT and HIF1α^−/−^ CD4^+^ naïve T cells activated under hypoxia for 48h were subjected to RNA-Seq. The gene expression analyses were performed using DESeq2 (version 1.34.0). The Wald test was used to calculate the p values and log2 fold changes. Genes with an adjusted p value < 0.05 and absolute log2 fold change > 1 were considered as differentially expressed genes (DEGs). A volcano plot was used to show all upregulated and downregulated DEGs using the ggplot2 R packagew (**D**), with top 50 identified DEGs shown as a heatmap (**E**). Top 10 enriched signaling pathways (downregulated) from Enriched Kyoto Encyclopedia of Genes and Genomes (KEGG) analyses of DEGs were shown in **F**. Significant terms of the KEGG pathways were selected with p value <0.05. **G**. mRNA expression of glycolytic genes was evaluated by real-time RT-PCR in T cells activated and cultured under normoxia (21%) and hypoxia (1%) for 48h. **H**. Protein expression of HIF1α, Glut1, Hk2, and Ldha was analyzed using cell lysates prepared using cells similarly activated as in **G**; β-actin was used as a loading control. **I.** WT naïve CD4^+^ T cells were similarly activated under normoxia and hypoxia as in **A** for 5.5 days -and analyzed for IFN-γ production. **J**. IFN-γ production by WT or VHL CD4^+^ T cells activated under hypoxia for 2.5 days. **K**. Naïve WT CD4^+^ T cells were similarly activated under hypoxia, in the presence of solvent (Control), 0.5 μM of 2-DG, or 0.2μM of DMOG, for 5.5 days, followed by analysis of IFN-γ production. L. Naive human CD4^+^ T cells isolated from PBMCs of healthy donors were activated under hypoxia for 5.5 days, with or without 0.5 μM 2-DG, followed by detection of IFN-γ. **M**. Equally mixed naïve CD45.1^+^ CD4^+^ T cells with naïve CD45.2^+^ CD4^+^ T cells from WT or HIF1α^−/−^ mice were activated under hypoxia for 5.5 days and detected for IFN-γ production. All the experiments were repeated at least twice. Pooled results shown in the dot plots and bar graphs depicted means ± SEM for all samples in each group, with each dot denoting an independent sample. **, p<0.01; ***, p<0.001; ****, p<0.0001. Source data were provided in the Source Data file.

Two early studies using human^32^ and mouse non-T_reg_ (CD4^+^CD25^−^) T cells^33^ show that hypoxia and HIF1α inhibit IFN-γ production. But because CD4^+^CD25^−^ T cells contain ~40–50% already-activated effector/central memory T cells (CD44^+^)^34,35^ that are equipped to produce IFN-γ, it remains unknown if and how HIF1α and hypoxia regulate IFN-γ induction in naïve T cells, upon activation. To address this, using the genetic mice that we previously generated with HIF1α specifically deleted in T cells (hereafter, HIF1α^−/−^)^8^, we isolated naïve CD4^+^ T cells from wildtype littermate controls (WT) and HIF1α^−/−^ mice and activated them with plate-bound antiCD3/CD28 and IL-2, in the presence and absence of IL-12. To directly compare the involvement of HIF1α in IFN-γ induction in normoxic and hypoxic T cells, these cells were cultured in a regular cell culture incubator (21% O_2_) and in a hypoxic chamber with 1% O_2_ mimicking hypoxic TME of solid tumors^25^ and inflammatory sites^26^. Although IL-12 is commonly used to drive optimal production of IFN-γ (T_H_1 differentiation) in activated T cells, we found that IFN-γ can be robustly induced without IL-12, albeit modestly reduced, as compared to IL-12-supplemented condition (Fig. S1A). Given this and to limit the confounding effects from IL-12 stimulation on IFN-γ induction, we primarily focused on the condition without IL-12, reasoning that this would allow us to more explicitly assess how T cell HIF1α regulates IFN-γ induction. Surprisingly, opposite to previous reports showing a negative role of HIF1α in IFN-γ production by CD4^+^CD25^−^ T cells^33^, HIF1α was essential for IFN-γ induction in naïve T cells, when activated under hypoxia (Fig. 1A). This was accompanied with substantial downregulation of T-bet (Fig. S1B), the master transcriptional regulator of IFN-γ expression. Consistent with our previous study^8^, IFN-γ induction in naïve T cells was unaffected by HIF1α deletion, under normoxia (Fig. 1B). To test if HIF1α also mediates IFN-γ induction in human naïve CD4^+^ T cells, upon activation under hypoxia, we knocked down human HIF1α using retroviruses expressing shRNAs against human HIF1α, which also led to significantly reduced IFN-γ induction (Fig. 1C). To extend this to CD8^+^ T cells, we sorted naïve CD8^+^ T cells from WT and HIF1α^−/−^ mice, similarly activated them under hypoxia, and analyzed their IFN-γ production. As shown in Fig. S1C, IFN-γ induction was reduced in HIF1α^−/−^ CD8^+^ T cells as well. These results together establish a selective role of HIF1α in IFN-γ induction in hypoxic but not normoxic T cells^8^.

To shed mechanistic light on this, we conducted whole transcriptome analysis (RNA-Seq) using total RNAs isolated from WT and HIF1α^−/−^ T cells that were activated, under normoxia and hypoxia for 48h. We chose this timepoint because it immediately precedes IFN-γ induction that occurs during the late-stage of T cell activation from Day 3–6, allowing us to establish a temporal relationship of HIF1α-mediated transcriptional and metabolic changes to subsequent IFN-γ induction. Also, activated T cells rapidly die around 56h after activation through AICD (see below), and 48h will maximize cell yield for downstream biochemical analyses. Whereas normoxic HIF1α^−/−^ T cells only displayed limited transcriptomic changes (600 hits) with 37 welldefined genes upregulated and 55 genes downregulated (DEGs: differentially expressed genes) (Fig. S1D), in sharp contrast, there were 5168 hits in hypoxic HIF1α^−/−^ T cells among which 583 were well-defined upregulated DEGs, and 399 were downregulated DEGs (Fig. 1D). This indicated a much more prominent impact of HIF1α in regulating transcriptomic programs in hypoxic T cells than in normoxic T cells. Notably, there was very little overlap among DEGs in HIF1α^−/−^ normoxic (Fig. S1E) vs hypoxic T cells (Fig. 1E), with top 50 genes shown in heatmaps, highlighting a rather distinct role of HIF1α in hypoxic T cells as compared to normoxic T cells. In further support, signaling pathway enrichment analyses using downregulated DEGs revealed minimal overlap as well (extended datasets, supplemental Table 1–2). Among the top 10 enriched downregulated pathways, HIF1α signaling pathway was the only shared hit between hypoxic T cells (Fig. 1F) and normoxic T cells (Fig. S1F). Similarly, there was barely any overlap among upregulated enriched pathways between HIF1α^−/−^ normoxic and hypoxic T cells (Fig. S1G, extended dataset, supplemental Table 1–2). A closer look at the other top 9 altered pathways in HIF1α^−/−^ hypoxic T cells showed that all of them are either directly involved in (#2, #3, #4, #7–10) or intimately interacted with cellular metabolism (#5 and #6), suggesting an essential role of HIF1α in orchestrating metabolic reprogramming in hypoxic T cells. In line with our previous report of a non-essential role of HIF1α in metabolic reprogramming in activated normoxic T cells^9^, none of these metabolic pathways appeared to be top hits in HIF1α^−/−^ normoxic T cells. Among all these highly intertwined metabolic processes (pentose phosphate pathway, central carbon metabolism, fructose and mannose metabolism, galactose metabolism, etc.), glycolysis was the most impacted metabolic pathway in hypoxic HIF1α^−/−^ T cells (#2) (Fig. 1F), with many of the most significantly altered genes being in this pathway (e.g., *Slc2a3*, *Slc16a3*, *Tpi1*, *Slc2a1*, *Eno1*, *Pkm*, *Hk2*, *Ldha*, *Gpi1*, *Gapdh*, *Aldoc*, etc., Fig. S1H). Although the role of glycolysis^7^, under the control of LDHa^36^ (the enzyme catalyzing the inter-conversion step of pyruvate to lactate in glycolysis) but not HIF1α^8^, has been reported in IFN-γ induction in normoxic T cells, whether HIF1α- glycolysis controls IFN-γ induction in hypoxic T cells is not defined.

To address this and inspired by the greatly inhibited IFN-γ induction and impacted glycolysis in hypoxic HIF1α^−/−^ T cells, we first directly confirmed the downregulated glycolytic pathway in HIF1α^−/−^ T cells by assessing mRNA expression of prototypical glycolytic molecules with realtime RT-PCR, all of which showed drastic downregulations in hypoxic HIF1α^−/−^ T cells (Fig. 1G). Although there were somewhat downregulations of these genes in normoxic HIF1α^−/−^ T cells as well, they were very modest, with quite a few being less than 2-fold (Fig. 1G), confirming a minor or no effect of HIF1α deletion on the glycolytic pathway in normoxic T cells, as indicated by our RNA-Seq analyses (Fig. S1F). Robust reductions of selected glycolytic molecules on protein level (i.e., Glut1-the major glucose transporter in T cells, hexokinase 2 (HK2)-a critical rate-limiting glycolytic enzyme catalyzing hexose phosphorylation, and LDHa) were also observed in hypoxic HIF1α^−/−^ T cells (Fig. 1H). We reasoned this was due to HIF1α stabilization by hypoxia (Fig. 1H) and thereby, HIF1α deletion left a more prominent impact on glycolysis in hypoxic T cells. To link HIF1α-glycolysis pathway to IFN-γ induction, we posit that hypoxic T cells with more potent HIF1α signaling and enhanced glycolysis^37^ would produce greater amount of IFN-γ than normoxic T cells, which was shown to be the case (Fig. 1I). As a complementary genetic gain-of-function (GOF) approach, we generated mice with conditional deletion of VHL in T cells, a primary negative regulator of HIF1α^28,29^ (hereafter, VHL^−/−^). Isolated naïve T cells from WT and VHL^−/−^ mice were similarly activated. As expected, VHL^−/−^ T cells harbored stabilized HIF1α and upregulated Glut1, a downstream targe of HIF1α. Like the functional GOF of HIF1α by hypoxia, genetic GOF of HIF1α by VHL deletion in T cells promoted IFN-γ production, as early as day 2 when minimal IFN-γ production can be detected in WT T cells, following activation (Fig. 1J). To pinpoint a role of glycolysis in this process, we treated T cells activated under hypoxia with 2-DG, a well-established glycolytic inhibitor. Clearly, 2-DG almost completely abolished IFN-γ induction in both mouse (Fig. 1K) and human T cells (Fig. 1L), pointing to an essential role of glycolysis in governing IFN-γ induction in hypoxic T cells. In contrast, DMOG, a cell permeable, competitive inhibitor of PHDs that stabilizes HIF1α, promoted IFN-γ induction (Fig. 1H). Lastly, to rule out the possibility of reduced IFN-γ in HIF1α^−/−^ T cells is due to differentially secreted factors, we mixed WT naïve CD45.1 T cells equally with either CD45.2 WT/ HIF1α^−/−^ naïve T cells, similarly activated under hypoxia, followed by detection of IFN-γ. As shown in Fig. 1M, introduction of WT CD45.1 T cells (and thus secreted factors by WT T cells) did not alter reduced IFN-γ induction in HIF1α^−/−^ T cells, suggesting this is a T cell-autonomous phenotype.

To gain a more complete picture of how HIF1α in T cell affects the effector function of hypoxic T cells upon activation, we analyzed other cytokines. Similar to reduced IFN-γ production, HIF1α^−/−^ hypoxic T cells produced significantly less amount of perforin (Prf) and granzyme (GzmB) (Fig. S1J), another two widely-regarded effector cytokines. Intriguingly, IL-2, a cytokine linked to early-stage of T cell activation^38^, was actually increased in HIF1α^−/−^ T cells (Fig. S1K), so was 2-DG-pretreated T cells (Fig. S1L), accentuating that HIF1α-glycolysis negatively regulates IL-2 production in activated T cells. Since IFN-γ, Prf, and GzmB are typically associated with latestage of T cell activation (note: they were barely detectable on Day 2–3, while abundant IL-2 can be detected at this time) (data not shown), these results unveil a reciprocal regulation of late vs early cytokines by HIF1α-glycolysis in hypoxic T cells, upon activation. In support of this conception, HIF1α stabilization by hypoxia (Fig. S1M) or VHL deletion in T cell (Fig. S1N) increased Prf and GzmB but decreased IL-2 production. Taken together, our study establishes HIF1α-aerobic glycolysis as a bona fide mediator of IFN-γ induction in hypoxic T cells.

### Direct regulation of IFN-γ induction by HIF1α and acetyl-CoA in hypoxic T cells

**Figure 2.**
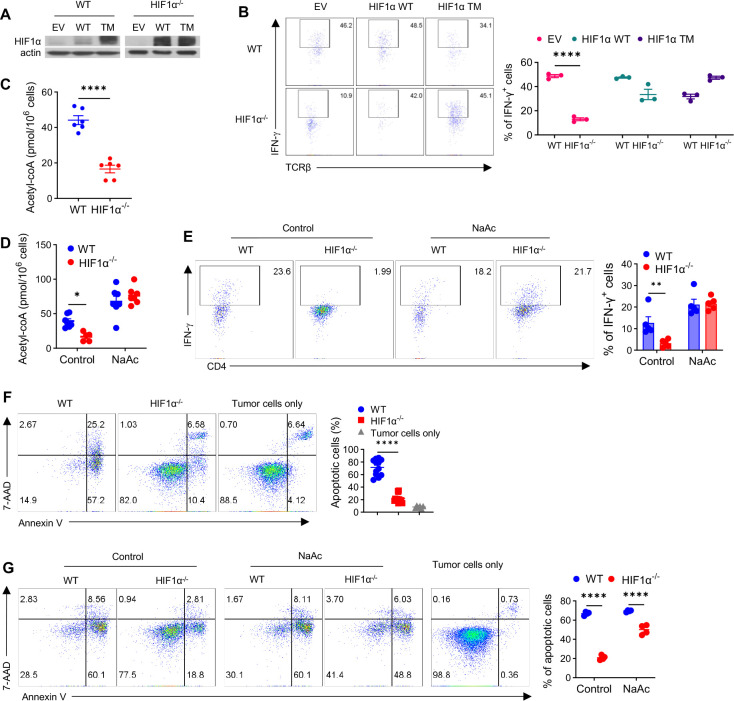
Direct regulation of IFN-γ induction in hypoxic T cells by HIF1α and [acetyl-CoA], *in vitro*. **A-B.** Protein expression of HIF1α in WT and HIF1α^−/−^ CD4^+^ T cells successfully transduced (GFP^+^) with empty retroviruses (EV) or retroviruses expressing WT or triple-mutant *HIF1α* (TM) (A). GFP^+^ T cells were activated under hypoxia and analyzed for IFN-γ production (**B**). **C.** [Acetyl-CoA] in activated WT and HIF1α^−/−^ CD4^+^ T cells. **D-E**. [Acetyl-CoA] (**D**) and IFN-γ production (**E**) by activated WT and HIF1α^−/−^ CD4^+^ T cells, with or without 20 mM sodium acetate (NaAc) added on Day 2 post-activation. **F**. Cell death of MB49 cells cultured alone (tumor cells only) or with activated WT and HIF1α^−/−^ CD4^+^ T cells at the ratio of 1:2 for 48h was measured by 7-AAD/Annexin V staining. **G**. Cell death of MB49 cells co-cultured with activated WT and HIF1α^−/−^ CD4^+^ T cells pretreated with or without NaAc for 48h was analyzed by 7-AAD/Annexin V staining. All experiments were repeated at least twice. Pooled results shown in the dot/bar graphs depicted means ± SEM for all the samples in each group, with each dot denoting an independent sample. **, p<0.01; ****, p<0.0001. Source data were provided in the Source Data file.

Our above results support a crucial role of T cell HIF1α-glycolysis in controlling IFN-γ induction in hypoxic T cells. We attempted to pinpoint a specific glycolytic mechanism, downstream of HIF1α, that drives IFN-γ induction in T cells, under hypoxia. To this end, we overexpressed individual key glycolytic molecules, i.e., Glut1, PKM2, LDHA, and MCT4, with a special focus on LDHa, considering an early study showing LDHa dictates IFN-γ induction in normoxic T cells^36^, However, successful overexpression of any of these individual molecules, including LDHa, in HIF1α^−/−^ hypoxic T cells (Fig. S2A) did not rescue impaired IFN-γ induction (Fig. S2B), arguing that this is unlikely mediated by individual glycolytic checkpoints but rather the whole-spectrum suppression of anaerobic glycolysis from the loss of HIF1α in T cells. To directly establish the role of HIF1α in inducing IFN-γ expression, we re-expressed HIF1α in HIF1α^−/−^ T cells, using two complementary approaches: overexpression of either WT HIF1α or hydroxylation-defective triple mutant HIF1α (P402A/P577A/N813A) that is stabilized and thereby remains constitutively active (HIF1α-TM)^39^. We achieved successful re-expression of HIF1α in hypoxic T cells with either WT HIF1α or HIF1α-TM (Fig. 2A). Importantly, this effectively re-stored IFN-γ production in HIF1α^−/−^ T cells to a level comparable to that in WT T cells (Fig. 2B). Together with the drastically reduced IFN-γ production in HIF1α^−/−^ T cells, these results highlight that HIF1α is not only necessary but also sufficient to drive IFN-γ induction in hypoxic T cells.

A direct consequence of the significantly decreased glycolytic activity is the depletion of intracellular pool of acetyl-CoA ([acetyl-CoA])^36,40^. Intrigued by a recent study showing that [acetyl-CoA] is instrumental for IFN-γ induction in normoxic LDHa^−/−^ T cells^36^, we asked if this is also responsible for impaired IFN-γ induction in hypoxic HIF1α^−/−^ T cells. To test this, we measured [acetyl-CoA] and found it was markedly reduced in hypoxic HIF1α^−/−^ T cells (Fig. 2C). Acetyl-CoA can be regenerated from acetate by acetyl-CoA synthetase independent of citrate release from mitochondria. We added acetate to the cultures of activated WT and HIF1α^−/−^ T cells on Day 2, prior to appreciable IFN-γ induction on Day 3. Cells were harvested on Day 5 to measure [acetyl-CoA] and detect IFN-γ production. As shown in Fig. 2D, acetate supplementation increased [acetyl-CoA] in HIF1α^−/−^ T cells to that of WT T cells. More importantly, this fully restored IFN-γ production in HIF1α^−/−^ T cells (Fig. 2E), supporting that the maintenance of [acetyl-CoA] in hypoxic T cells by HIF1α-anaerobic glycolysis is a major metabolic mechanism underpinning IFN-γ induction. Since IFN-γ has long been known to be essential for anti-tumor responses^41–43^, we evaluated the ability of HIF1α^−/−^ T cells to kill tumor cells by co-culturing activated WT or HIF1α^−/−^ hypoxic T cell with MB49 bladder tumor cells at a ratio of 2:1. Cell death of MB49 cells was analyzed 48h later by staining for Annexin V and 7AAD, two commonly used markers for detection of early vs late apoptotic cells. As shown in Fig. 2F, HIF1α^−/−^ T cells were less able to kill tumor cells, as compared to WT T cells (Fig. 2F).

When T cells harvested from these co-cultures were analyzed for their IFN-γ production, HIF1α^−/−^ T cells produced much less IFN-γ (Fig. S2C). Because acetate supplementation re-installed IFN-γ production in HIF1α^−/−^ T cells (Fig. 2E), we asked if this could restore their tumor-killing capacity. Indeed, acetate-pretreated HIF1α^−/−^ T cells had greatly improved ability to kill co-cultured MB49 tumor cells, approaching that of WT T cells (Fig. 2G). Collectively, these data show that HIF1α, by controlling the anaerobic glycolysis and sustaining [acetyl-CoA], orchestrates effector function and tumor-killing ability of hypoxic T cells.

### Impaired IFN-γ induction in HIF1α^−/−^ T cells is not due to their proliferative defect.

**Figure 3.**
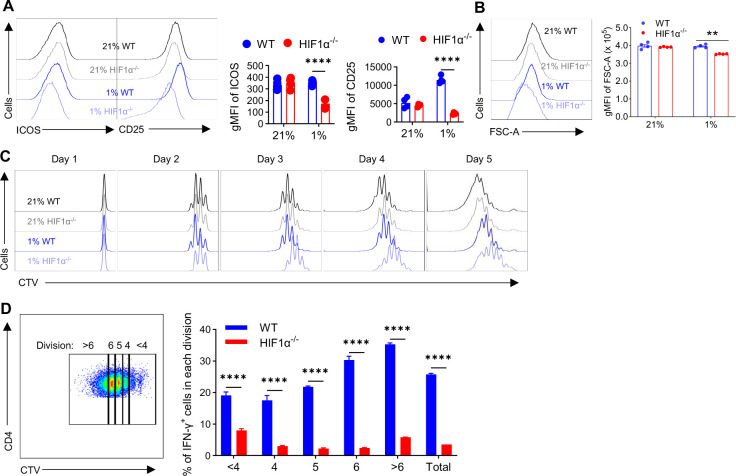
Reduced IFN-γ production in HIF1α^−/−^ T cells is not due to proliferative defect. **A-B**. Naïve WT and HIF1α^−/−^ CD4^+^ T cells were activated under normoxia (21% O_2_) and hypoxia (1% O_2_) for 5.5 days. Gated live cells were analyzed for the expression of ICOS and CD25 (**A**), depicted as geometric mean fluorescence intensity (gMFI), and area of forward scatter (FSC) (**B**). **C-D**. Naïve WT and HIF1α^−/−^ CD4^+^ T cells were labeled with CellTrace Violet (CTV) and activated under normoxia (21% O_2_) and hypoxia (1% O_2_). CTV dilution was monitored daily to assess cell proliferation. Gating strategy on defining cell division by CTV dilution was shown for WT T cells (left) and IFN-γ production by activated WT and HIF1α^−/−^ CD4^+^ T cells within indicated cell divisions was shown by the bar graph on the right (**D**). All the experiments were repeated at least twice. Pooled results shown in the dot plots and bar graphs depicted means ± SEM for all the samples in each group, with each dot denoting an independent sample. **, p<0.01; ****, p<0.0001. Source data were provided in the Source Data file.

Next, we want to explore cellular mechanisms underlying the blocked IFN-γ induction in hypoxic HIF1α^−/−^ T cells. Since we previously showed that glycolysis is an essential component of metabolic reprogramming during T cell activation^9^, and hypoxic HIF1α^−/−^ T cells had drastically reduced glycolysis, we posit that their activation would be substantially impaired. To test this, we employed two widely used markers to assess T cell activation: inducible T-cell COStimulator (ICOS) and CD25. Consistent with a largely dispensable role of HIF1α in the metabolic reprogramming in activated T cell under normoxia^9^, we did not see overtly altered expression of ICOS and CD25 in normoxic HIF1α^−/−^, as compared to WT T cells; in stark contrast, their expression was greatly reduced in hypoxic HIF1α^−/−^ T cells vs WT T cells (Fig. 3A). Another cardinal feature of less active T cells is that they are smaller in size^44^, which can be measured by forward scatter (FSC). As shown in Fig. 3B, in keeping with selectively reduced activation of hypoxic HIF1α^−/−^ T cells, only hypoxic but not normoxic HIF1α^−/−^ T cells were smaller than their WT counterparts. To link T cell activation to the glycolytic activity in hypoxic T cells, we analyzed ICOS and CD25 expression in glycolysis^high^ (Glut1^high^) vs glycolysis^low^ (Glut1^low^) T cells, which showed significant reductions in both CD4^+^Glut1^low^(Fig. S3A) as well as CD8^+^ Glut1^low^ T cells (Fig. S3B), in comparison to their Glut1^high^ counterparts. Since CD25 is also regarded as a T_reg_ marker, we ruled out the possibility that reduced CD25 expression in activated hypoxic HIF1α^−/−^ CD4^+^ T cells was due to the different abundance of iT_reg_ by staining for FoxP3, which appeared comparable (Fig. S3C). These data together showed that HIF1α deletion in hypoxic T cells, by decreasing their glycolytic activity, leads to selectively impaired T cell activation.

As known, T cell activation initiates a series of intracellular events, engaging a multitude of signaling cascades and biochemical processes that drive T cell proliferation. As such, in line with the impaired activation of hypoxic HIF1α^−/−^ T cells, we observed much slower proliferation, indicated by significantly reduced Ki-67 Fig. (S3D), a widely used marker for proliferative cells. To more explicitly examine this, we stained WT and HIF1α^−/−^ naïve CD4^+^ T cells with CellTrace Violet (CTV), whose dilution can distinctively label each division of cell proliferation. CTVlabeled cells were activated under normoxia and hypoxia. Consistent with unaltered activation of normoxic HIF1α^−/−^ T cells, their proliferation appeared normal, whereas a significant delayed proliferation of hypoxic HIF1α^−/−^ CD4^+^ T cells was observed. This defect was evident as early as day 2 (Fig. 3C), shortly after the initial T cell growth phase (~24h)^9^. Given the reported intimate relationship of cell proliferation and IFN-γ production^45^, we asked if the proliferative defect in hypoxic HIF1α^−/−^ T cells could be responsible for reduced IFN-γ production, by directly comparing IFN-γ production in WT and HIF1α^−/−^ T cells within the same division. As shown in Fig. 3D and S3E, HIF1α^−/−^ T cells exhibited reduced IFN-γ production, regardless of their division, indicating proliferative defect in HIF1α^−/−^ CD4^+^ T cells is not a main cellular mechanism.

### HIF1α-glycolysis-driven AICD controls IFN-γ induction in hypoxic T cells.

**Figure 4.**
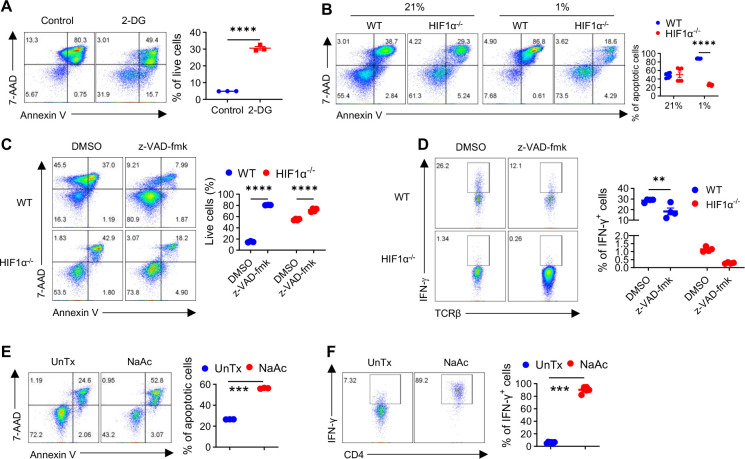
HIF1α-glycolysis-driven AICD governs IFN-γ production in hypoxic T cells. **A**. Cell death of naïve WT CD4^+^ T cells activated under hypoxia for 3 days, with or without with 2-DG was measured by 7-AAD/Annexin V staining. **B**. Cell death of naïve WT and HIF1α^−/−^ CD4^+^ T cells activated under normoxia (21% O_2_) and hypoxia (1% O_2_) for 3 days was detected by 7-AAD/Annexin V staining. **C-D**. Naïve WT and HIF1α^−/−^ CD4^+^ T cells were activated under hypoxia, with z-VAD-fmk or without (DMSO); on day 3, cells were stained for 7-AAD/Annexin V to assess cell death (**C**), and on day 5, IFN-γ production was determined (**D**). **E-F**. Naïve HIF1α^−/−^ CD4^+^ T cells were activated under hypoxia, with or without 20mM sodium acetate (NaAc) added on day 0; on day 3, cells were stained for7-AAD and Annexin V to assess cell death (**E**), and IFN-γ production was determined on day 5 (**F**). All the experiments were repeated at least twice. Pooled results shown in the dot plots depicted means ± SEM for all the samples in each group, with each dot denoting an independent sample. **, p<0.01; ***, p<0.001; ****, p<0.0001. Source data were provided in the Source Data file.

Another major outcome following T cell activation is AICD, which is commonly regarded as a housekeeping process to remove obsolete effector T cells during the contraction phase after a successful immune response. When gone awry, this would disrupt the immune homeostasis, causing autoimmune diseases^46^ and/or breaching transplantation tolerance^47^. However, whether AICD governs the formation of effector T cells (e.g., IFN-γ producing T_H_1 cells) is unknown. Considering the weaker activation of hypoxic HIF1α^−/−^ T cells, we contemplated that this would dampen AICD. To gain a complete idea of this dynamic process, we conducted a time-course study, wherein apoptosis of activated WT and HIF1α^−/−^ T cells was measured by staining for Annexin V and 7-AAD, the main form of cell death underpinning AICD^47^. While not much cell death was observed on Day 1 and Day 2 following T cell activation, it rapidly arose on Day 3 in WT T cells (approximately 56h post-activation), and very few cells remained alive (only ~9%, Fig. S4A); this process was considerably delayed in HIF1α^−/−^ T cells, with > 40% of cells staying alive on Day 3. Similar trends persisted on Day 4 and Day 5. Because HIF1α^−/−^ T cells were less metabolically active and hence consumed less nutrients in the medium, we wondered if the improved survival of HIF1α^−/−^ T cells was simply because they had more nutrients to support their survival. To test this, we replaced half of the old media with fresh media daily and found that this did not change the inhibited AICD in hypoxic HIF1α^−/−^ T cells (Fig. S4B), arguing against a role of differential nutritional statuses of the cultures in this process. In addition, since activated HIF1α^−/−^ T cells produce more IL-2 that has long been known as a growth factor for T cells^47^, we wondered if this would enable HIF1α^−/−^ T cells to survive better. We blocked IL-2 with an effective neutralizing anti-IL2 antibody that we previously used^48^, which did not rectify inhibition of AICD in HIF1α^−/−^ T cells (Fig. S4C). Furthermore, given an established role of IFN-γ in driving AICD^49^, we wondered if reduced IFN-γ in hypoxic HIF1α^−/−^ T cells could contribute to this phenotype. We thus added recombinant IFN-γ to WT and HIF1α^−/−^ T cell activated under hypoxia. As shown in Fig. S4D, even high doses of exogenous IFN-γ did not rescue the delay of AICD in HIF1α^−/−^ T cells. Together, these results suggest that inhibited AICD in hypoxic HIF1α^−/−^ T cells is an unlikely outcome of altered extrinsic factors but rather a direct consequence of intrinsic alterations (e.g., decreased glycolytic activity). In support of this, glycolytic inhibition with 2-DG blocked AICD and improved survival of hypoxic T cells (Fig. 4A) upon activation. Similar effect of 2-DG was also observed in hypoxic human T cell (Fig. S4E).

We hypothesized that impaired AICD in HIF1α^−/−^ hypoxic T cells may drive reduced IFN-γ production. In support of this hypothesis, first, we observed a direct correlation of selectively reduced IFN-γ induction and impaired AICD in hypoxic HIF1α^−/−^ T cells (Fig. 4B), whereas in normoxic T cells there was no impairment of ACID and therefore, no reduction of IFN-γ production. Second, we directly assess the role of AICD in IFN-γ induction by treating hypoxic T cells with z-VAD-fmk, a cell-permeable, irreversible pan-caspase inhibitor that blocks all features of apoptosis and AICD^47^. As expected, z-VAD-fmk substantially improved the survival of activated WT T cells (Fig. 4C) and importantly, this markedly reduced IFN-γ production in WT hypoxic T cells (Fig. 4D), strongly supporting a positive role of AICD in driving IFN-γ induction in hypoxic T cells. Moreover, despite the already very low level of IFN-γ produced by HIF1α^−/−^ T cells, z-VAD-fmk further reduced this (Fig. 4D), in line with a modest yet significant suppression of AICD in HIF1α^−/−^ T cells. Third, considering that acetate supplementation rescued impaired IFN-γ induction in hypoxic HIF1α^−/−^ T cells, we wondered if this was mediated by re-engaging AICD. To test this, we activated HIF1α^−/−^ T cells under hypoxia, with or without acetate supplementation, followed by detection of AICD on Day 3 and IFN-γ production on Day 5. Clearly, acetate supplementation greatly increased AICD in HIF1α^−/−^ T cells (Fig. 4E) and concomitantly, augmented IFN-γ production (Fig. 4F). Taken together, these results indicate that HIF1α-glycolysis-acetyl CoA drives AICD in hypoxic T cells and induces IFN-γ production, lacking which led to impaired AICD and IFN-γ induction.

### Loss of HIF1α selectively impaired IFN-γ production in TILs

**Figure 5.**
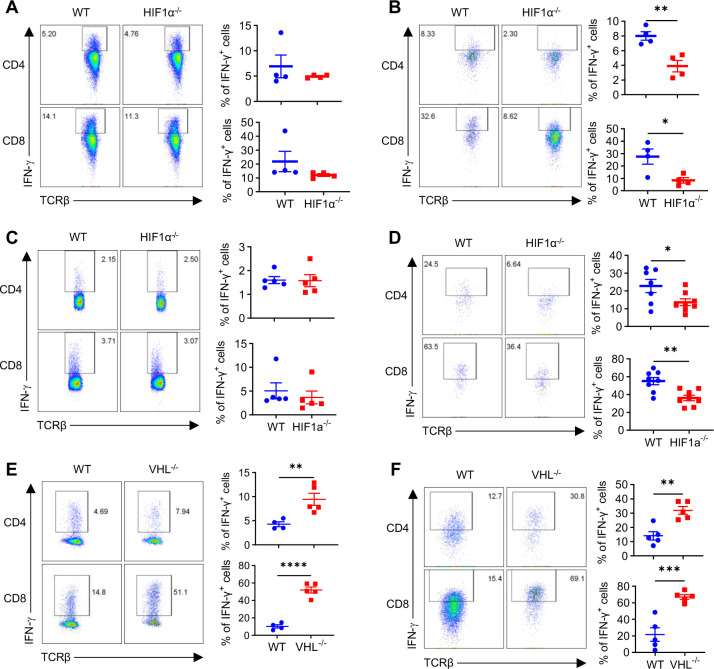
HIF1α regulates IFN-γ production in tumor-infiltrating T cells (TILs). **A-B**. T cells isolated from WT or HIF1α^−/−^ mice bearing established MB49 bladder tumor were analyzed for IFN-γ production by CD4^+^ and CD8^+^ splenocytes (**A**) or TILs (**B**). **C-D**. T cells isolated from WT or HIF1α^−/−^ mice bearing established orthotopic B16-BL6 melanoma were analyzed for IFN-γ production by CD4^+^ and CD8^+^ splenocytes (**C**) or TILs (**D**). **E-F**. T cells isolated from WT or VHL^−/−^ mice bearing established MB49 bladder tumor were analyzed for IFN-γ production by CD4^+^ and CD8^+^ splenocytes (**E**) or TILs (**F**). All the experiments were repeated 2–5 times. Pooled results shown in the dot plots depicted means ± SEM for all the mice in each group, with each dot denoting an independent sample. **, p<0.01; ***, p<0.001; ****, p<0.0001. Source data were provided in the Source Data file.

Having demonstrated a selective role of HIF1α in controlling IFN-γ induction in hypoxic T cells *in vitro*, we wanted to recapitulate this *in vivo*. As known, TME in solid tumors is a highly hypoxic milieu, with O_2_ levels being ~1%^25^; on the other hand, peripheral lymphoid organs, like spleens and draining lymph nodes (DLNs), represent more oxygenated environments, although their O_2_ tensions are also below 21%^20,21^. We reason that T cells from spleens/DLNs and TILs form a natural, albeit not ideal, *in vivo* system to evaluate how HIF1α controls IFN-γ production in T cells under normoxia vs hypoxia. To this end, we inoculated WT and HIF1α^−/−^ mice with MB49 bladder tumor cells. Once established tumors formed, tumor-bearing mice were euthanized to harvest TILs and T cells from spleens and DLNs, followed by analyses of IFN-γ production. Whereas there was no overt reduction of IFN-γ in HIF1α^−/−^ CD4^+^ and CD8^+^ T cells from spleen (Fig. 5A) and DLN (Fig. S5A), it was substantially decreased in HIF1α^−/−^ TILs (Fig. 5B), as compared to their WT counterparts, corroborating our *in vitro* data showing a selective role of HIF1α in driving IFN-γ production in hypoxic T cells. Likewise, the production of GzmB (Fig. S5B) and Prf (Fig. S5C) was significantly lower in HIF1α^−/−^ TILs but not T cells from spleens and DLNs than their corresponding WT T cells. Also, in parallel to greater production of IFN-γ, GzmB, and Prf by hypoxic T cells over normoxic T cells *in vitro*, TILs also produced those cytokines at a higher level than T cells from spleens (Fig. S5D). Intrigued by these results from the transplantable MB49 bladder tumor model, we wanted to further confirm this using an orthotopic tumor model. To this end, we employed the well-established B16-BL6 melanoma that can be established by intradermally injecting syngeneic B16-BL6 cells into B6 mice. Isolated TILs and splenocytes were similarly analyzed, as aforementioned. Consistent with what we observed in the MB49 bladder tumors, IFN-γ production was also highly comparable between HIF1α^−/−^ CD4^+^ and CD8^+^ splenocytes and their WT counterparts isolated from B16 melanomabearing mice (Fig. 5C); a significant reduction was seen in HIF1α^−/−^ CD4^+^ and CD8^+^ TILs as compared to WT TILs (Fig. 5D). Taken together, HIF1α is selectively required for IFN-γ production in hypoxic TILs but not more oxygenated peripheral T cells.

Our *in vitro* assays showed that VHL^−/−^ T cells with stabilized HIF1α were predisposed to produce IFN-γ as early as day 2.5, when activated under hypoxia (Fig. 1F). To determine if VHL deletion in T cells would also promote IFN-γ production *in vivo*, we inoculated WT and VHL^−/−^ mice with MB49 tumor cells. Mice with established tumors were euthanized to harvest TILs and T cells from spleens and DLNs. As expected and consistent with our *in vitro* data, we observed increased expression of Glut1 in all VHL^−/−^ T cells harvested from spleen, DLNs, and tumors (Fig. S5E), as a result of stabilized HIF1α in VHL^−/−^ T cells^8^. In parallel to our *in vitro* results, there was a greater IFN-γ production in hypoxic VHL^−/−^ TILs than WT TILs (Fig. 5F). Noteworthily, because HIF1α stabilization occurs in peripheral VHL^−/−^ T cells, we saw significantly increased IFN-γ production even in these oxygenated T cells from spleen (Fig. 5E) and DLNs (Fig. S5F). Overall, these in *vivo* results comply with our *in vitro* data, establishing a pivotal role of HIF1α in controlling IFN-γ production in T cells, when it gets stabilized by hypoxia (e.g., TME) or deletion of its negative regulator (e.g., VHL).

### HIF1α in T cells governs ICB efficacy.

**Figure 6.**
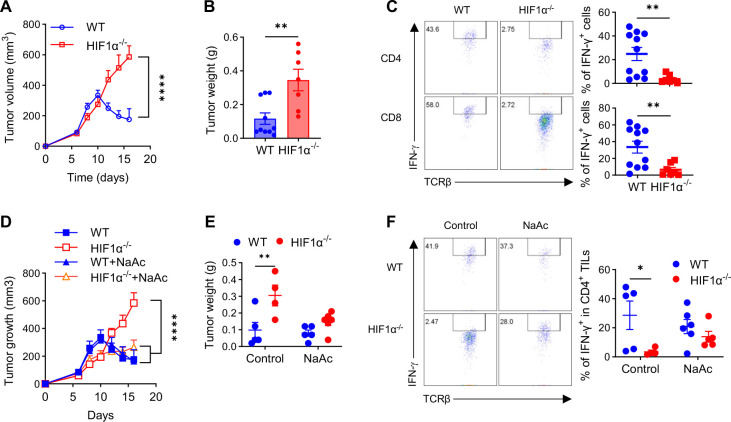
HIF1α in T cells governs therapeutic effects of ICBs. **A-B**. WT and HIF1α^−/−^ mice bearing palpable MB49 bladder tumor were treated with combined anti-CTLA-4+anti-PD-1 therapy, followed by periodic measurement of tumor volume (**A)**; upon euthanization, tumors were weighed (**B**). **C**. IFN-γ production by CD4^+^ and CD8^+^ TILs isolated from tumor-bearing mice in **A-B**. **D-E**. WT and HIF1α^−/−^ mice bearing palpable MB49 bladder tumor were treated with combined anti-CTLA-4+anti-PD-1 alone or in conjunction with administration of sodium acetate (NaAc), followed by periodic measurement of tumor volume (**D)**; upon euthanization, tumors were weighed (**E**). **F**. IFN-γ production by CD4^+^ TILs from tumor-bearing mice in **D-E**. All the experiments were repeated 2–5 times. Pooled results shown in the dot plots depicted means ± SEM for all the mice in each group, with each dot denoting a mouse. *, p<0.05; **, p<0.01; ****, p<0.0001. Source data were provided in the Source Data file.

ICBs have emerged as a major pillar of cancer care ^50–55^. While functional rejuvenation (e.g. IFN-γ production) of TILs by ICBs has been known to be important for their efficacy^41,43,56^, specific underlying mechanisms are not well-understood. Our results show that HIF1α in T cells is instrumental for the effector function of TILs. We therefore asked if HIF1α in T cells would represent an essential metabolic and molecular mechanism governing therapeutic effects of ICBs. To test this, we treated WT and HIF1α^−/−^ mice bearing palpable MB49 bladder tumors with combined anti-CTLA-4+anti-PD-1 (combo), a more efficacious ICB therapy than monotherapies (anti-CTLA-4 or anti-PD-1 alone)^57^, following the regimen that we previously described^41^. As shown in Fig. 6A, while combo potently suppressed tumor growth in WT mice, it failed to do so in HIF1α^−/−^ mice, indicating a pivotal role of T cell HIF1α in dictating ICB efficacy. We euthanized these mice on day 18 and measured their tumor weights. Tumors from combotreated WT mice were visually smaller and weighed significantly less than those from combotreated HIF1α^−/−^ mice (Fig. 6B). When isolated T cells from these mice were analyzed for their IFN-γ production, we observed a selective reduction of IFN-γ in HIF1α^−/−^ TILs (Fig. 6C) but not T cells from spleen (Fig. S6A) or DLNs (Fig. S6B), as compared to their WT counterparts, again confirming a specific role of HIF1α in hypoxic T cells. Our results are consistent with an early study showing that HIF1α in CD8^+^ T cells is required for optimal anti-tumor immunity in colorectal cancer (MC38)^58^, following adoptive T-cell therapy (another major form of immunotherapy) and ICBs. Together, these studies support an important role of T cell-intrinsic HIF1α signaling in mediating anti-tumor immunity and ICB efficacy in various types of tumor.

A significant barrier in ICBs is the therapeutic resistance to these novel therapeutics ^57,59^. Our above results, together with a previous study^58^, inform loss of the HIF1α signaling pathway in T cells as a major mechanism of ICB resistance. However, how to overcome this ICB resistance has been elusive, a solution to which would undoubtedly expand the clinical utilization of ICBs and benefit more patients. Inspired by our data showing that acetate supplementation can rescue reduced IFN-γ production and impaired tumor killing ability of HIF1α^−/−^ T cells *in vitro*, we asked if this could act as an effective strategy to circumvent ICB resistance in HIF1α^−/−^ tumorbearing mice, *in vivo*. To this end, we administered acetate to tumor-bearing WT and HIF1α^−/−^ mice, followed by combo ICB treatments. While acetate supplementation alone did not improve combo efficacy in WT tumor-bearing mice, remarkably, this led to greatly improved therapeutic effects of combo in HIF1α^−/−^ mice, evidenced by greatly suppressed tumor growth (Fig. 6D), reduced tumor weights (Fig. 6E), and significantly increased IFN-γ production by HIF1α^−/−^ CD4^+^ (Fig. 6F) and CD8^+^ (Fig. S6C) TILs.

Putting everything together, we show that the HIFa signaling in T cells, by maintaining their glycolytic activity (hence, [acetyl-CoA]) and AICD, drives IFN-γ induction. Loss of HIF1α in T cells impairs their tumor killing capacity and renders tumor-bearing mice resistant to ICBs, which can be reversed by acetate supplementation. Considering that GTA is approved to treat infants with Canavan disease (an indication of highly acceptable toxicity profile), we envision a smooth translation of our findings, which can lead to a rapid repurposing of GTA as an effective therapeutic intervention for ICB resistance, a pressing unmet medical need.

## Discussion

Cellular metabolism emerges as the fundamental driving force in T cell activation and differentiation^5–15^, by supporting their drastically increased bioenergetic and biosynthetic demands^16^. Despite glycolysis is a key component of this metabolic reprogramming associated with T cell activation and T_H_1 differentiation^7,36^, under normoxia, this is largely independent of T cell HIF1α but rather, actively controlled by Myc^8,9^. Given this, it remains unknown whether and how T cell HIF1α regulates IFN-γ induction/T_H_1 differentiation, under hypoxia, and whether this relies on anaerobic glycolysis. Considering hypoxia is a common feature in various phathophysiological contexts, especially in the TME of solid tumors, the lumen of the GI tract, and inflammatory sites, a clear understanding of this is importance. Using T cell-specific GOF and LOF genetic systems as well as pharmacological modulators, we unveil an indispensable role of T cell HIF1α signaling in inducing IFN-γ production in hypoxic but not normoxic T cells, upon activation, in a process dependent on AICD but not activation-driven cell proliferation. Severely impaired anaerobic glycolysis in hypoxic HIF1α^−/−^ T cells results in depleted [acetyl-CoA], and replenishment of [acetyl-CoA] with acetate supplementation restores IFN-γ production in HIF1α^−/−^ hypoxic T cells. Similarly, hypoxic HIF1α^−/−^ TILs but not oxygenated peripheral T cells from spleens and DLNs have reduced IFN-γ production. Most importantly, HIF1α^−/−^ mice bearing established tumors are resistant to ICBs, which can be treated with acetate supplementation, providing an effective strategy to overcome ICB resistance.

To the best of our knowledge, this is the first study to show that HIF1α is a bona fide regulator of IFN-γ induction in hypoxic T cells, supported by drastically reduced IFN-γ production in HIF1α^−/−^ T activated under hypoxia (*in vitro*) and HIF1α^−/−^ TILs (*in vivo*). Conversely, HIF1α stabilization in hypoxic T cells, TILs, and VHL^−/−^ T cells significantly increased IFN-γ induction over normoxic T cells, oxygenated peripheral T cells, and T cells from littermate control mice, respectively. Moreover, re-expression of HIF1α-WT or constitutively active HIF1α-TM in hypoxic HIF1α^−/−^ T cells largely restores their capacity to produce IFN-γ. IFN-γ induction in hypoxic T cells is completely dependent on their glycolytic activity, as glycolytic inhibition with 2-DG abolishes it. Correlatively, HIF1α deletion in hypoxic T cells severely downregulates glycolysis and greatly depletes [acetyl-CoA]. As such, acetate supplementation, by replenishing [acetyl-CoA], restores IFN-γ production in HIF1α^−/−^ T cells both *in vitro* and *in vivo*. Interestingly, production of other late effector cytokines (e.g., Prf and GzmB), in addition to IFN-γ, is also reduced in hypoxic HIF1α^−/−^ T cells. In contrast, production of an early cytokine IL-2^38^ is increased in HIF1α^−/−^ T cells, pointing to a reciprocal regulatory role of HIF1α in late vs early cytokine production. Our results seem to conflict with two previous studies^32,33^ wherein a negative role of hypoxia and HIF1α in IFN-γ production in CD4^+^CD25^−^ was reported. However, it is noteworthy to mention that CD4^+^CD25^−^ cells contain ~40–50% of already activated effector/central memory T cells (CD44^+^)^34,35^ that are capable of producing IFN-γ. While this discrepancy may be due to the differential effect of HIF1α and hypoxia in IFN-γ production by already-activated T cells vs IFN-γ induction in naïve T cells (being activated), this warrants further investigations. That said, our results are in line with previous reports showing that specific deletion of VHL in T_reg_ cells^60^ led to increased IFN-γ production, and that VHL deletion^61^ or PHD deletion^62^ in T cells promoted the polyfunctionality of CD8^+^ T cells (i.e., increased production of IFN-γ, TNF, and GzmB), and that total (not naïve) CD8^+^ T cells activated under hypoxia also express higher levels of IFN-γ^63^. Interestingly, although we show that HIF1α is dispensable for IFN-γ induction^8^ and aerobic glycolysis in normoxic T cells^9^, a recent study reported that T cell LDHa maintains aerobic glycolysis and [acetyl-CoA] in normoxic T cells, promoting IFN-γ induction and T_H_1 differentiation^36^. Here, we find that HIF1α plays an essential role in IFN-γ induction in hypoxic T cells, by maintaining anaerobic glycolysis and [acetyl-coA], which is largely independent of LDHa, as overexpression of LDHa did not rescue the impaired IFN-γ induction in HIF1α^−/−^ hypoxic T cells. While this can be explained to certain extent that LDHa is just one of many glycolytic genes regulated by HIF1α in hypoxic T cells, these studies otherwise highlight that LDHa but not HIF1α is required for IFN-γ induction in normoxic T cells, whereas HIF1α but not LDHa is essential for IFN-γ induction in hypoxic T cells, although they both exert this function by maintaining glycolysis and [acetyl-CoA]. To this end, we show that glycolytic inhibition with 2-DG almost completely shuts down IFN-γ induction in both normoxic^8^ and hypoxic T cells (this study).

We previously showed that glycolysis is an essential component of metabolic reprogramming during T cell activation under normoxia, which is under primary regulation of Myc but not HIF1α^9^. Acute deletion of HIF1α in normoxic T cells only led to minor or no impact on glycolysis and as such, no overt changes to cell growth, cell activation, and activation-driven cell proliferation were found in HIF1α^−/−^ T cells^9^, consistent with our results reported here. In contrast, under hypoxia, HIF1α deletion in T cells impacts a multitude of metabolic processes, encompassing central carbon metabolism (#3 hit), fructose and mannose metabolism (#4 hit), galactose metabolism (#7 hit), and pentose phosphate pathway (#8 hit), in addition to glycolysis (#2 hit), suggesting HIF1α is an important mediator of metabolic reprogramming in hypoxic T cells. It would be interesting to dissect out the differential roles of HIF1α and Myc in orchestrating metabolic reprogramming in T cell activated under normoxia vs hypoxia, in the near future. Accompanied with reduced glycolysis pathway in hypoxic HIF1α^−/−^ T cells is impaired T cell activation that in turn leads to suppressed AICD and cell proliferation defect. Interestingly, we find that impaired AICD but not delayed cell proliferation in HIF1α^−/−^ hypoxic T cells contributes to reduced IFN-γ induction, as IFN-γ reduction persists in all HIF1α^−/−^ cells, regardless of cell division; conversely, inhibition of AICD substantially reduces IFN-γ production in WT T cells and even decreases the already low level of IFN-γ production in HIF1α^−/−^ T cells. We largely ruled out inhibition of AICD in HIF1α^−/−^ hypoxic T cells is mediated by secreted extrinsic factors, as refreshing half of the old medium daily, blocking IL-2, and adding IFN-γ to HIF1α^−/−^ T cell cultures could not rescue this phenotype. Our results support that this is cell-autonomous outcome from decreased glycolysis and reduced [acetyl-CoA], as replenished [acetyl-CoA] by acetate supplementation re-engages AICD in HIF1α^−/−^ hypoxic T cells, leading to their restored IFN-γ production and tumor killing capacity. Of note, AICD has long been considered as a housekeeping process to eliminate unwanted effector T cells, a byproduct of a successful immune response. In return, this saves space for useful T cells and maintain immune homeostasis. When blocked, pathologies such as autoimmunity^46^ and loss of transplantation tolerance^47^ arise. Our data on the other hand strongly argue for an active role of AICD in promoting IFN-γ induction in hypoxic T cells, regulated by HIF1α-glycolysis-acetyl-CoA axis. Because the Fas-FasL axis^46^ is important in mediating AICD in T cells, future studies ought to assess how HIF1α cross talks with this axis.

Unprecedented therapeutic effects of ICBs in various types of late-stage cancer have propelled immunotherapy as a mainstay therapy for cancer patients^50–55^. However, their efficacy has reached a plateau. For instance, even with the combination therapy of anti-CTLA-4 and anti-PD-1, only ~34% of patients with advanced melanoma exhibit progression-free survival^57^. Functional rejuvenation of TILs by ICBs^41,43,56^ has been dubbed as a major mechanism underlying ICB efficacy. How to effectively boost the effector function of TILs (e.g., IFN-γ production) would be key to expand the clinical successes of ICBs. Considering that TILs and tumor cells co-exist in a metabolically challenging milieu characterized by hypoxia and poor nutrition, substantial research endeavors in recent years have been devoted to studying the intratumoral metabolic tug-of-war between TILs and tumor cells, in order to tilt it in favor of TILs. To this end, an early study showed that glycolysis^high^ tumors, by consuming glucose, suppress the effector function of TILs, which can be reversed by ICBs (i.e., anti-CTLA-4, anti-PD-1/L1)^64^. However, a more recent study found that anti-CTLA-4 was only effective in glycolysis^low^ tumors, which the authors attributed to the preferential feeding of glucose to T_reg_, leading to phenotypic and functional destabilization of intratumoral T_reg_ toward IFN-γ and TNF-producing effector T cells^65^. Despite the discrepancy between these two studies, it is clear that glycolysis is intimately involved in shaping TILs. Our study shows that dysfunctional HIF1α-anaerobic glycolysis in TILs significantly impairs effector function of TILs, including IFN-γ production, rendering tumor-bearing mice resistant to ICBs. Together with a previous study using other tumor models, we argue that T cell-intrinsic loss of HIF1α is a major mechanism of therapeutic resistance to ICBs. Most importantly, we show that administration of acetate to HIF1α^−/−^ mice bearing established tumors can re-sensitize them to ICBs, manifesting an effective strategy to overcome this ICB resistance, a pressing unmet medical need in the clinic. Interestingly, another recent study^66^ reported that Warburg effect in tumor cells (reliance on glycolysis) is mainly mediated by the diversion of pyruvate flux away from acetyl-CoA generation; by replenishing [acetyl-CoA], acetate supplementation reverses Warburg effect and drive tumor cell differentiation, which in turn suppresses tumorigenesis. This, together with the functional boosting effects of acetate supplementation on TILs after ICBs, highlight acetate supplementation as an ideal “two birds, on stone” strategy to boost anti-tumor responses. Given that glycerol triacetate (GTA), a form of acetate supplementation, is already approved by the FDA to treat infant with Canavan disease, our results justify further clinical testing of GTA as a repurposed medicine for ICB resistance.

In summary, we find that HIFa-glycolysis, by maintaining [acetyl-CoA] and activating AICD, drives IFN-γ induction selectively in hypoxic T cells. Specific deletion of HIF1α in T cells largely abolishes T cells’ ability to kill tumor cells and tumor-bearing mice’s responses to ICBs. We further demonstrate that this can be recovered by acetate supplementation (Fig. S6D).

## Methods

### Mice and cell lines

The HIF1α^−/−^ mice with specific HIF1α deletion in T cells were generated by crossing floxed HIF1α mice (Jackson Laboratory, Stock No.: 007561) with CD4-Cre transgenic mice (Jackson Laboratory, Stock No.: 022071)^8^. VHL^−/−^ mice with VHL specifically deleted in T cells were similarly generated by crossing floxed VHL mice (Jackson Laboratory, Stock No.: 012933) with CD4-Cre transgenic mice. CD45.1 mice on B6 background (Jackson Laboratory, Stock No.: 002014) were procured from The Jackson Laboratory (Bar Harbor, ME) and bred in our animal facility. All mice were housed in specific pathogen-free conditions in the animal facility of The University of Alabama at Birmingham (UAB) under 12 hours/12 hours light/dark cycle, ambient room temperature (22 °C) with 40%−70% humidity. Seven to twelve-week-old mice were used in the experiments. Male mice were used for MB49 model, as MB49 tumor cell line was generated in a male mouse to avoid cross-sex immune response. To facilitate random assignment of mice inoculated with B16-BL6 melanoma cells to different groups, we used female mice, as adult male mice typically show aggressive behaviors and could confound the experiments with uneven distribution of tumor sizes. Our animal protocol (APN-21945) was approved by Institutional Animal Care and Use Committee at UAB. All tumor-bearing mice were humanely euthanized prior to their tumors reaching the maximally allowed tumor size (20 mm in diameter) in our animal protocol. The MB49 bladder cells were kindly provided by Dr. A. Kamat at MD Anderson Cancer Center. MB49 cells were cultured in DMEM supplemented with 10% FBS and 100 units/mL of penicillin and 100 μg/mL of streptomycin (all from Invitrogen). The B16-BL6 murine melanoma cells were kindly provided by Dr I. Fidler at MD Anderson Cancer Center and cultured with MEM supplemented with 10% FBS, 2mM L-glutamine, 1mM sodium pyruvate, 1% non-essential amino acids, 1% vitamin, 100 units/mL of penicillin and 100 μg/mL of streptomycin (all from Invitrogen). All cells were cultured in a humidified 37 °C incubator with 5% CO_2_. All cells were regularly tested with the MycoAlert detection kit (Lonza, LT07–118) to ensure they were free of mycoplasma contamination.

### Mouse naïve T cell isolation and activation

Naïve T cells were isolated from spleens and lymph nodes by negative selection using microbeads following the manufacturer’s instructions (Miltenyi, #130–104-453 for CD4 and #130096-543 for CD8). The purity of CD4^+^CD62L^hi^CD44^lo^CD25^−^ naïve CD4 T cells and CD8^+^CD62L^hi^CD44^lo^ naïve CD8 T cells were confirmed by flow cytometry. Freshly isolated naïve CD4 T cells were stimulated with plate-bound anti-CD3 (Clone 145–2C11, Bio X cell, #BE0001–1) and anti-CD28 (Clone 37.51, Bio X cell, #BE0015–1) in presence of 100U/mL human IL-2. Plates were pre-coated with 2μg/mL anti-CD3 and anti-CD28 for at least 2h. Naïve T cells were activated with plate-bond anti-CD3 plus anti-CD28 in presence of 100U/mL IL-2, with 0.2ng/mL or without IL-12. For hypoxic condition, cultures were placed in a hypoxic chamber with oxygen level set at 1% to mimick the O_2_ tension in the TME of solid tumors. In some experiments, 50% of old media were refreshed with freshly prepared Click’s medium daily. Where designated, 20mM of NaAc, 0.5 μM of 2-DG (Sigma, #8375), 0.2 μM of DMOG (Sigma, #400091) or solvent control was added to the culture medium. Cell apoptosis and transcription factors were checked on day 2.5 and cytokines were check on day 5.5 after a brief PMA and ionomycin stimulation, as we described before^8^. For *in vitro* CD45.1/CD45.2 co-culture, CD45.1 naïve CD4 T cells were equally mixed with WT or HIF1α^−/−^ naïve CD4^+^ T cells (congenically labeled with CD45.2). Cells were then activated and IFN-γ was checked after PMA and inonomycin stimulation. To block IL-2, cells were similarly activated, in the presence of anti-mouse IL-2 (Bio X Cell, #BE0043) at 10μg/mL. To assess T cell proliferation, cells were pre-labeled with 2 μM CellTrace Violet (CTV, Thermo Fisher, #C34557) by incubating for 20 min with periodical mixing. After incubation, cells were washed twice with complete culture medium to remove soluble CTV. To set up co-culture of hypoxic T cells with MB49 cells, we activated WT or HIFα^−/−^ T cells for 5 days under hypoxia and co-cultured with MB49 tumor cells at the ratio of T cell (effector): MB49 (target) of 2:1. The culture plate was pre-coated with 0.2 μg/mL of anti-CD3 for 2h. Cell apoptosis of tumor cells and IFN-γ production in T cells was analyzed 48h later.

### Human naïve CD4 T cell isolation and activation, 2-DG treatment

Human naïve CD4 T cells were isolated from Leukocyte Reduction System (LRS) Cones (procured from Lifesouth community blood centers). Total PBMC cells were flushed from human LRS cone with phosphate-buffered saline (PBS) containing 2% fetal bovine serum (FBS). Centrifuge at 800 × g for 10 minutes before the lysis of red blood cells. Human naïve CD4 T cells were purified by negative selection using microbeads following the manufacturer’s manual (Miltenyi, #130–094-131). The purity was more than 95% checked by flow cytometry. Freshly isolated naïve CD4 T cells were activated with plate-bound anti-human CD3 (Clone UCHT1, BioLegend, #300438) and anti-human CD28 (Clone CD28.2, BioLegend, #302934) in the presence of 100 U/mL human IL-2. 0.5 μM of 2-DG or solvent control was added on day 0. To knock down human *HIF1Α* using shRNAs, we designed and synthesized the oligoes from IDT and cloned it to retroviral vector LMP. The target sequences of human shHIF1α are sh#2 GGGTTGAAACTCAAGCAACTG. The inserts were validated by DNA sequencing. Retroviruses were packed by co-transfection of transfer plasmid and packaging plasmid pMD2.G to phoenix cells. Retroviral particles were then used to transduce activated human T cells using spin-infection approach as for mouse T cells (described below).

### Plasmid construction, virus packaging and transduction

The coding sequences (CDS) of murine *Hif1α*, *Glut1*, *Pkm2*, *Ldha* and *Mct4* were PCR-amplified from mouse first-strand cDNA library produced by reverse transcription (Invitrogen, #11752–050) and cloned into the retroviral vector pMIG II. The inserts were validated by DNA sequencing. Retrovirus was produced by co-transfection of phoenix cells with transfer plasmid and packaging plasmid pCL-Eco. Virus-containing culture medium was collected at 48 and 72h post-transfection. Freshly isolated naïve CD4 T cells were activated for 24h before retroviral spin-infection in virus-containing culture medium with the presence of Lipofectamine 3000 (Invitrogen, #L3000–150) and human IL-2, as we previously described^67^. Successfully transduced T cells expressing GFP were sorted by flow cytometry. The overexpression of interested genes was validated by western blot. To check IFN-γ production, sorted GFP^+^ cells were re-activated on 0.2 μg/mL anti-CD3 pre-coated plate for 4 days under hypoxia.

### Cell apoptosis assay

Cell apoptosis was analyzed by flow cytometry, as described before^68^. Naïve T cells were activated under specified normoxic or hypoxic conditions. On day 2.5, cells were harvested, washed once with DPBS and again with 1× Annexin V binding buffer before staining with Annexin V (1:50, Thermo Fisher, #17–8007) and 7-AAD (1:200, Sigma, #129935) or propidium iodide (1:50, Thermo Fisher, #00–6990-42) in 1× Annexin V binding buffer for 30 min at room temperature. Cells were acquired by Attune NxT Flow Cytometer and data were analyzed by FlowJo.

### *In vivo* tumor inoculation and treatment

Mice were shaved on the right flank one day before tumor inoculation. On day 0, anesthetized mice were inoculated subcutaneously with 5 × 10^5^ of MB49 cells to the right flank. Anti-CTLA-4 (Bio X Cell, clone 9H10) and anti-PD-1 (Bio × Cell, clone 29F.1A12) or isotype controls was given by intraperitoneal injection (i.p.) on day 6, 9, and 12 at a dose of 200, 100, and 100 μg per mouse, respectively, as we previously described^41^. To establish orthotopic B16-BL6 melanoma model, anesthetized mice were inoculated intradermally with 1.25 × 10^5^ of B16-BL6 cells into the right flanks on day 0. Tumors were measured by caliper every other day starting from day 6 and tumor volumes (mm^3^) were calculated using the formula (0.52× length × width^2^). The tumor-bearing mice were sacrificed at indicated time points. Upon euthanization, tumors, tumor-draining lymph nodes and spleens were collected, and tumor weights were recorded. For NaAc treatment *in vivo*, mice were treated with 500 mg/kg i.p. plus drinking water containing 200mM sodium acetate starting from day 3. Control mice were injected with equal volume of DPBS at the same time and fed with regular drinking water.

### TILs isolation, tumor-draining lymph nodes (dLN) and splenocyte preparation

Tumors were collected into ice-cold RPMI 1640 containing 2% FBS and minced into fine pieces on ice, followed by digestion with 400 U/mL collagenase D (Worthington Biochemical Corporation, #LS004186) and 20 μg/mL DNase I (Sigma, #10104159001) at 37 °C for 40 min with periodic mixing. EDTA (Sigma, #1233508) was then added to the final concentration of 10 mM to stop digestion. Cell suspensions were filtered through 70 μM cell strainers, and TILs were obtained by collecting the cells in the interphase after Ficoll (MP Biomedicals, #091692254) separation. Spleens and tumor-draining lymph nodes were collected in ice-cold HBSS containing 2% FBS to prepare single cell suspensions. Cells were filtered through 70 μM nylon mesh after lysis of red blood cells. TILs, dLNs and splenocytes were all re-suspended in complete Click’s medium (Irvine Scientific, #9195–500mL) for following staining and flow cytometric analyses.

### Flow cytometric analysis

For surface staining, single cell suspensions were incubated with antibody cocktails in DPBS containing 2% (wt/vol) BSA for 30 min on ice. LIVE/DEAD^™^ Fixable Aqua Dead Cell Stain Kit (Thermo Fisher: L34957) was performed according to the manufacturer’s instructions. To stain transcriptional factors, cells were fixed by the fixation buffer in the FoxP3/Transcription Factor Staining Buffer Set (Invitrogen, #00–5523-00) following surface staining, and stained intracellularly according to the manufacturer’s instructions. To detect intracellular cytokines, cells were briefly stimulated for 4–5 h with phorbol 12-myristate 13-acetate (PMA, final concentration: 50 ng/mL; Sigma, #P8139–5MG) plus ionomycin (final concentration: 1 μM; Sigma, #I0634–1MG) in the presence of monensin (BD Biosciences, #51–2092KZ). Stimulated cells were stained with surface markers, then fixed using the fixation buffer in the BD Cytofix/Cytoperm Plus Fixation/Permeabilization Kit (BD Biosciences, #554715), and stained for cytokines with corresponding antibodies ntracellularly, according to the manufacturer’s instructions. Used flow Antibodies are listed in the Supplemental Table. All the flow cytometric data were acquired using the built-in software of the Attune NxT Flow Cytometer (Invitrogen, A24860) from Thermo Fisher. Flow cytometric data were analyzed using FlowJo (version 10.8.1).

**Table:** 

Antibodies	Clone	Source	Identifier	Antibody dilution

Anti-Mouse CD8 Brilliant Violet 785^™^	53-6.7	BD Biosciences	#563332	1/200
Anti-Mouse CD4 Brilliant Violet 421^™^	RM4-5	BioLegend	#100544	1/200
Anti-Mouse CD45 PerCP-Cyanine5.5	30-F11	Thermo Fisher	#45-0451-82	1/200
Anti-Mouse CD45.1 PerCP-Cyanine5.5	A20	BioLegend	#110728	1/200
Anti-Mouse CD45.2 Alexa Fluor^®^ 700	104	BioLegend	#109822	1/200
Anti-Mouse TCRβ APC-Cy7	H57-597	BioLegend	#109220	1/200
Anti-Mouse Perforin PE	S16009A	BioLegend	#154306	1/100
Anti-Mouse Granzyme B FITC	QA16A02	BioLegend	#372206	1/100
Anti-Mouse IFN-γ Brilliant Violet 650^™^	XMG1.2	BioLegend	#505832	1/100
Anti-Mouse IL-2 Brilliant Violet 711^™^	JES6-5H4	BioLegend	#503837	1/100
Anti-Mouse T-bet Brilliant Violet 711^™^	4B10	BioLegend	#644820	1/100
Anti-Mouse ICOS PerCP-Cyanine5.5	7E.17G9	BioLegend	#117424	1/200
Anti-Mouse CD25 APC	PC61.5	Thermo Fisher	#17-0251-82	1/200
Anti-Mouse FoxP3 eFluor^™^ 450	FJK-16s	Thermo Fisher	#48-5773-82	1/100
Anti-Mouse HIF1α APC	Mgc3	Thermo Fisher	#17-7528-82	1/100
Anti-Mouse Ki-67 Alexa Fluor^®^ 700	SolA15	Thermo Fisher	#56-5698-82	1/100
Anti-Human/Mouse Glut1 Alexa Fluor^®^ 405	EPR3915	Abcam	#ab210438	1/100
Anti-Human CD3 Super Bright^™^ 702	OKT3	Thermo Fisher	#67-0037-42	1/200
Anti-Human IFN-γ Brilliant Violet 605^™^	B27	BD Biosciences	#562974	1/100
Anti-Human CD4 Brilliant Violet 650^™^	L200	BD Biosciences	#563737	1/200

### Western blot (WB)

Western blot was performed, as previously described^69^. Briefly, cells were washed with cold DPBS twice before lysed with M-PER buffer (Thermo Scientific, #78501) containing proteinase inhibitors cOmplete (Roche, #11836170001) and phosphatase inhibitors (Sigma, #P2850 and P5726). Lysates were then collected and transferred to 1.5 mL Eppendorf tubes and sonicated. Protein concentration was determined by NanoDrop. Fifty μg of total proteins were loaded onto each lane of an 8–12% SDS-PAGE gel. After electrophoresis, proteins on the gel were transferred to 0.45 μm of PVDF membrane (Millipore, #IPVH00005) in a sponge sandwich. Membranes were then blocked with 5% of non-fat milk (Bio-Rad, #170–6404) and probed with primary antibodies overnight on a shaker in cold room. Membranes were then washed and incubated with HRP-conjugated secondary antibodies for 1 h at room temperature. The membranes were then incubated with Western HRP substrate (Millipore, WBLUR0500) for 2–5 min before imaging with an X-ray film. The antibodies used for WB are listed in Supplemental Table. β-actin was blot as loading control on the same gel with proteins of interest. Uncropped and unprocessed scans of all blots were provided in the Source Data file.

**Table:** 

Name	Clone	Source	Catalog	Antibody dilution

HIF-1α	D2U3T	Santa Cruz Biotechnology	14179S	1/1000
Glut1	polyclonal	Sigma	07-1401	1/1000
Hk2	C64G5	Cell Signaling Technology	#2867	1/1000
Ldha	Polyclonal	Cell Signaling Technology	2012S	1/1000
Pkm2	D78A4	Cell Signaling Technology	4053T	1/1000
Mct4	D-1	Santa Cruz Biotechnology	sc-376140	1/1000
β-Actin	C-4	Santa Cruz Biotechnology	sc-47778 HRP	1/10000

### Acetyl-CoA measurement

Naïve CD4 T cells were differentiated under hypoxia for 4 days before cultured in fresh Click’s medium for another 24h with or without 20mM sodium acetate. Cells were lysed with M-PER buffer for 10 minutes on ice. Cell lysates were spun down at 13000 rpm for 10min at 4 °C. Supernatant was deproteinized using 4M perchloric acid and neutralized by 1N potassium hydroxide. Then deproteinized lysates were used for acetyl-coA measurement using Acetyl-Coenzyme A Assay Kit (Sigma, #MAK039) following the manufacturer’s instructions. Fluorescence intensity was measured at Ex/Em=535/587nM. Acetyl-coA levels were normalized to cell number.

### RT-PCR

Total RNAs were extracted from cells using RNeasy Plus Mini kit (QIAGEN, #74136) according to the manufacturer’s instructions. Reverse transcription polymerase chain reaction (RT-PCR) was done, as we previously described^48^. In brief, first-strand cDNAs were synthesized by SuperScript III reverse transcriptase (Invitrogen, # 11752250) according to the manufacturer’s instructions. Up to 1μg of total RNA was used for reverse transcription. First-strand cDNA was diluted 20 times for quantitative RT-PCR which was performed on Bio-Rad CFX96 instrument. The primers are listed in Supplemental Table. β-actin was used as the housekeeping gene. Specificity of primers were all validated by single peak of melting curve. The gene expression level was calculated using the 2^−ΔΔCT^ method.

**Table:** 

Gene name	Forward primer	Reverse primer

Hif1α	AGCTTCTGTTATGAGGCTCACC	TGACTTGATGTTCATCGTCCTC
Glut1	CAGTTCGGCTATAACACTGGTG	GCCCCCGACAGAGAAGATG
Hk2	TGATCGCCTGCTTATTCACGG	AACCGCCTAGAAATCTCCAGA
Gpi	TCAAGCTGCGCGAACTTTTTG	GGTTCTTGGAGTAGTCCACCAG
Tpi1	CCAGGAAGTTCTTCGTTGGGG	CAAAGTCGATGTAAGCGGTGG
Pkm2	GCCGCCTGGACATTGACTC	CCATGAGAGAAATTCAGCCGAG
Eno1	TGCGTCCACTGGCATCTAC	CAGAGCAGGCGCAATAGTTTTA
Ldha	CATTGTCAAGTACAGTCCACACT	TTCCAATTACTCGGTTTTTGGGA
Mct4	TCACGGGTTTCTCCTACGC	GCCAAAGCGGTTCACACAC
Actin	CATTGCTGACAGGATGCAGAAGG	TGCTGGAAGGTGGACAGTGAGG

### RNA-seq analysis

Naïve CD4 T cells isolated from WT or HIF1α^CD4^ mice were activated for 48h under hypoxia. Cells were directly lysed on the plate and total RNA was extracted immediately by RNeasy Plus Mini Kit from QIAGEN, Inc. Standard RNA-seq was performed by GENEWIZ, Inc. Briefly, total RNA was enriched with Poly A selection and sequencing was performed on Illumina platform. For RNA-seq data analysis, paired-end transcriptome sequences were mapped to the *Mus musculus* GRCm38 reference genome available on ENSEMBL using the STAR aligner (version 2.7.5a. Read counts per gene were calculated using htseq-count in the HTseq package (version 0.11.2)^70^. Then the read counts per gene were used for downstream differential gene expression analysis and pathway enrichment analysis. The analysis of differentially expressed genes (DEGs) between WT control and HIF1α^−/−^ samples was performed using DESeq2 (version 1.34.0)^71^ in R (version 3.6.0). The Wald test was used to calculate the p-values and log2 fold changes. Genes with an adjusted p-value <0.05 and absolute log2 fold change >1 were considered as DEGs. A volcano plot was used to show all upregulated and downregulated DEGs using the ggplot2 package (version 3.3.6) (ggplot2: Elegant Graphics for Data Analysis. Springer-Verlag New York. ISBN 978–3-319–24277-4, https://ggplot2.tidyverse.org). Enriched Kyoto Encyclopedia of Genes and Genomes (KEGG) pathways^72^ of the DEGs were identified by enrichr package^73^ (version 3.0), a comprehensive gene set enrichment analysis tool. Significant terms of the KEGG pathways were selected with a p-value <0.05.

### Statistical analysis

For animal experiments, 5–7 mice were included in each group; for *in vitro* studies with cells, triplicates were set up to ensure consistency and reproducibility. All experiments were repeated for 2–5 times. Representative results were expressed as mean ± SEM. Data were analyzed using a two-sided Student’s t-test, one-way ANOVA, or two-way ANOVA after confirming their normal distribution. All analyses were performed using Prism 9.4.0 (GraphPad Software, Inc.) and p < 0.05 was considered statistically significant.

## Supplementary Material

Supplement 1

## Data Availability

The RNA-seq data generated in this study have been deposited in the Gene Expression Omnibus (GEO) database under accession code GSE [https://www.ncbi.nlm.nih.gov/geo/query/acc.cgi?acc=GSE]. Deposited data are publicly available. The remaining data in this study are available within the manuscript or Supplementary Data, with source data provided herein. Source data are provided with this paper.

## References

[1] Mosmann T. R., Cherwinski H., Bond M. W., Giedlin M. A. & Coffman R. L. Two types of murine helper T cell clone. I. Definition according to profiles of lymphokine activities and secreted proteins. J Immunol 136, 2348–2357 (1986). 2419430

[2] Harrington L. E. Interleukin 17-producing CD4+ effector T cells develop via a lineage distinct from the T helper type 1 and 2 lineages. Nat Immunol 6, 1123–1132, doi:10.1038/ni1254 (2005). 16200070

[3] Sakaguchi S., Sakaguchi N., Asano M., Itoh M. & Toda M. Immunologic self-tolerance maintained by activated T cells expressing IL-2 receptor alpha-chains (CD25). Breakdown of a single mechanism of self-tolerance causes various autoimmune diseases. J Immunol 155, 1151–1164 (1995). 7636184

[4] Shen H. & Shi L. Z. Metabolic regulation of TH17 cells. Mol Immunol 109, 81–87, doi:10.1016/j.molimm.2019.03.005 (2019). 30903829 PMC7059830

[5] Chang C. H. Posttranscriptional control of T cell effector function by aerobic glycolysis. Cell 153, 1239–1251, doi:10.1016/j.cell.2013.05.016 (2013). 23746840 PMC3804311

[6] Ho P. C. Phosphoenolpyruvate Is a Metabolic Checkpoint of Anti-tumor T Cell Responses. Cell 162, 1217–1228, doi:10.1016/j.cell.2015.08.012 (2015). 26321681 PMC4567953

[7] Michalek R. D. Cutting edge: distinct glycolytic and lipid oxidative metabolic programs are essential for effector and regulatory CD4+ T cell subsets. J Immunol 186, 3299–3303, doi:10.4049/jimmunol.1003613 (2011). 21317389 PMC3198034

[8] Shi L. Z. HIF1αlpha-dependent glycolytic pathway orchestrates a metabolic checkpoint for the differentiation of TH17 and Treg cells. J Exp Med 208, 1367–1376, doi:10.1084/jem.20110278 (2011). 21708926 PMC3135370

[9] Wang R. The transcription factor Myc controls metabolic reprogramming upon T lymphocyte activation. Immunity 35, 871–882, doi:10.1016/j.immuni.2011.09.021 (2011). 22195744 PMC3248798

[10] Pearce E. L. Enhancing CD8 T-cell memory by modulating fatty acid metabolism. Nature 460, 103–107, doi:10.1038/nature08097 (2009). 19494812 PMC2803086

[11] van der Windt G. J. Mitochondrial respiratory capacity is a critical regulator of CD8+ T cell memory development. Immunity 36, 68–78, doi:10.1016/j.immuni.2011.12.007 (2012). 22206904 PMC3269311

[12] Ma E. H. Serine Is an Essential Metabolite for Effector T Cell Expansion. Cell metabolism 25, 482, doi:10.1016/j.cmet.2017.01.014 (2017). 28178570

[13] Mondanelli G. A Relay Pathway between Arginine and Tryptophan Metabolism Confers Immunosuppressive Properties on Dendritic Cells. Immunity 46, 233–244, doi:10.1016/j.immuni.2017.01.005 (2017). 28214225 PMC5337620

[14] Hu X. Synthetic RORgamma agonists regulate multiple pathways to enhance antitumor immunity. Oncoimmunology 5, e1254854, doi:10.1080/2162402X.2016.1254854 (2016). 28123897 PMC5215247

[15] Bensinger S. J. LXR signaling couples sterol metabolism to proliferation in the acquired immune response. Cell 134, 97–111, doi:10.1016/j.cell.2008.04.052 (2008). 18614014 PMC2626438

[16] Pearce E. L. Metabolism in T cell activation and differentiation. Curr Opin Immunol 22, 314–320, doi:10.1016/j.coi.2010.01.018 (2010). 20189791 PMC4486663

[17] Chi H. Regulation and function of mTOR signalling in T cell fate decisions. Nat Rev Immunol 12, 325–338, doi:10.1038/nri3198 (2012). 22517423 PMC3417069

[18] Zenewicz L. A. Oxygen Levels and Immunological Studies. Front Immunol 8, 324, doi:10.3389/fimmu.2017.00324 (2017). 28377771 PMC5359232

[19] Habler O. P. & Messmer K. F. The physiology of oxygen transport. Transfus Sci 18, 425–435, doi:10.1016/s0955-3886(97)00041-6 (1997). 10175156

[20] Caldwell C. C. Differential effects of physiologically relevant hypoxic conditions on T lymphocyte development and effector functions. J Immunol 167, 6140–6149, doi:10.4049/jimmunol.167.11.6140 (2001). 11714773

[21] Cho S. H. Germinal centre hypoxia and regulation of antibody qualities by a hypoxia response system. Nature 537, 234–238, doi:10.1038/nature19334 (2016). 27501247 PMC5161594

[22] Brandtzaeg P. Immunobiology and immunopathology of human gut mucosa: humoral immunity and intraepithelial lymphocytes. Gastroenterology 97, 1562–1584, doi:10.1016/0016-5085(89)90406-x (1989). 2684725

[23] Karhausen J. Epithelial hypoxia-inducible factor-1 is protective in murine experimental colitis. J Clin Invest 114, 1098–1106, doi:10.1172/jci21086 (2004). 15489957 PMC522241

[24] Carreau A., El Hafny-Rahbi B., Matejuk A., Grillon C. & Kieda C. Why is the partial oxygen pressure of human tissues a crucial parameter? Small molecules and hypoxia. J Cell Mol Med 15, 1239–1253, doi:10.1111/j.1582-4934.2011.01258.x (2011). 21251211 PMC4373326

[25] Palazon A., Aragones J., Morales-Kastresana A., de Landazuri M. O. & Melero I. Molecular pathways: hypoxia response in immune cells fighting or promoting cancer. Clin Cancer Res 18, 1207–1213, doi:10.1158/1078-0432.CCR-11-1591 (2012). 22205687

[26] Colgan S. P. & Taylor C. T. Hypoxia: an alarm signal during intestinal inflammation. Nat Rev Gastroenterol Hepatol 7, 281–287, doi:10.1038/nrgastro.2010.39 (2010). 20368740 PMC4077542

[27] Semenza G. L. Targeting HIF-1 for cancer therapy. Nat Rev Cancer 3, 721–732, doi:10.1038/nrc1187 (2003). 13130303

[28] Maxwell P. H. The tumour suppressor protein VHL targets hypoxia-inducible factors for oxygen-dependent proteolysis. Nature 399, 271–275, doi:10.1038/20459 (1999). 10353251

[29] Tanimoto K., Makino Y., Pereira T. & Poellinger L. Mechanism of regulation of the hypoxia-inducible factor-1 alpha by the von Hippel-Lindau tumor suppressor protein. Embo j 19, 4298–4309, doi:10.1093/emboj/19.16.4298 (2000). 10944113 PMC302039

[30] Lando D., Peet D. J., Whelan D. A., Gorman J. J. & Whitelaw M. L. Asparagine hydroxylation of the HIF transactivation domain a hypoxic switch. Science 295, 858–861, doi:10.1126/science.1068592 (2002). 11823643

[31] Zhu J. & Paul W. E. CD4 T cells: fates, functions, and faults. Blood 112, 1557–1569, doi:10.1182/blood-2008-05-078154 (2008). 18725574 PMC2518872

[32] Bollinger T. HIF-1alpha- and hypoxia-dependent immune responses in human CD4+CD25high T cells and T helper 17 cells. Journal of leukocyte biology 96, 305–312, doi:10.1189/jlb.3A0813-426RR (2014). 24664971

[33] Shehade H., Acolty V., Moser M. & Oldenhove G. Cutting Edge: Hypoxia-Inducible Factor 1 Negatively Regulates Th1 Function. J Immunol 195, 1372–1376, doi:10.4049/jimmunol.1402552 (2015). 26179900

[34] Marusina A. I. CD4(+) virtual memory: Antigen-inexperienced T cells reside in the naïve, regulatory, and memory T cell compartments at similar frequencies, implications for autoimmunity. Journal of autoimmunity 77, 76–88, doi:10.1016/j.jaut.2016.11.001 (2017). 27894837 PMC6066671

[35] Beyer M. & Schultze J. L. CD4+CD25highFOXP3+ regulatory T cells in peripheral blood are primarily of effector memory phenotype. Journal of clinical oncology : official journal of the American Society of Clinical Oncology 25, 2628–2630; author reply 2630–2622, doi:10.1200/jco.2006.08.0192 (2007). 17577047

[36] Peng M. Aerobic glycolysis promotes T helper 1 cell differentiation through an epigenetic mechanism. Science 354, 481–484, doi:10.1126/science.aaf6284 (2016). 27708054 PMC5539971

[37] Cho S. H. Hypoxia-inducible factors in CD4(+) T cells promote metabolism, switch cytokine secretion, and T cell help in humoral immunity. Proc Natl Acad Sci U S A 116, 8975–8984, doi:10.1073/pnas.1811702116 (2019). 30988188 PMC6500120

[38] Coyle A. J. The CD28-related molecule ICOS is required for effective T cell-dependent immune responses. Immunity 13, 95–105 (2000). 10933398 10.1016/s1074-7613(00)00011-x

[39] Lee J. H., Elly C., Park Y. & Liu Y. C. E3 Ubiquitin Ligase VHL Regulates Hypoxia-Inducible Factor-1α to Maintain Regulatory T Cell Stability and Suppressive Capacity. Immunity 42, 1062–1074, doi:10.1016/j.immuni.2015.05.016 (2015). 26084024 PMC4498255

[40] Moussaieff A. Glycolysis-mediated changes in acetyl-CoA and histone acetylation control the early differentiation of embryonic stem cells. Cell metabolism 21, 392–402, doi:10.1016/j.cmet.2015.02.002 (2015). 25738455

[41] Shi L. Z. Interdependent IL-7 and IFN-gamma signalling in T-cell controls tumour eradication by combined alpha-CTLA-4+alpha-PD-1 therapy. Nat Commun 7, 12335, doi:10.1038/ncomms12335 (2016). 27498556 PMC4979067

[42] Fu T., He Q. & Sharma P. The ICOS/ICOSL pathway is required for optimal antitumor responses mediated by anti-CTLA-4 therapy. Cancer Res 71, 5445–5454, doi:0008-5472.CAN-11-1138 [pii] (2011). 21708958 10.1158/0008-5472.CAN-11-1138

[43] Curran M. A., Montalvo W., Yagita H. & Allison J. P. PD-1 and CTLA-4 combination blockade expands infiltrating T cells and reduces regulatory T and myeloid cells within B16 melanoma tumors. Proc Natl Acad Sci U S A 107, 4275–4280, doi:10.1073/pnas.0915174107 (2010). 20160101 PMC2840093

[44] Yang K. T cell exit from quiescence and differentiation into Th2 cells depend on Raptor-mTORC1-mediated metabolic reprogramming. Immunity 39, 1043–1056, doi:10.1016/j.immuni.2013.09.015 (2013). 24315998 PMC3986063

[45] Setoguchi R., Matsui Y. & Mouri K. mTOR signaling promotes a robust and continuous production of IFN-γ by human memory CD8+ T cells and their proliferation. Eur J Immunol 45, 893–902, doi:10.1002/eji.201445086 (2015). 25476730

[46] Nagata S. Human autoimmune lymphoproliferative syndrome, a defect in the apoptosis-inducing Fas receptor: a lesson from the mouse model. J Hum Genet 43, 2–8, doi:10.1007/s100380050029 (1998). 9609991

[47] Wells A. D. Requirement for T-cell apoptosis in the induction of peripheral transplantation tolerance. Nat Med 5, 1303–1307, doi:10.1038/15260 (1999). 10545998

[48] Shi L. Z. Inhibitory role of the transcription repressor Gfi1 in the generation of thymus-derived regulatory T cells. Proc Natl Acad Sci U S A 110, E3198–3205, doi:10.1073/pnas.1300950110 (2013). 23918371 PMC3752244

[49] Refaeli Y., Van Parijs L., Alexander S. I. & Abbas A. K. Interferon gamma is required for activation-induced death of T lymphocytes. J Exp Med 196, 999–1005, doi:10.1084/jem.20020666 (2002). 12370261 PMC2194022

[50] Shen H. Predictive biomarkers for immune checkpoint blockade and opportunities for combination therapies. Genes & Diseases (2019). 10.1016/j.gendis.2019.06.006 PMC6997608 32042863

[51] Cella D. Patient-reported outcomes of patients with advanced renal cell carcinoma treated with nivolumab plus ipilimumab versus sunitinib (CheckMate 214): a randomised, phase 3 trial. The lancet oncology 20, 297–310, doi:10.1016/S1470-2045(18)30778-2 (2019). 30658932 PMC6701190

[52] Antonia S. J. Durvalumab after Chemoradiotherapy in Stage III Non-Small-Cell Lung Cancer. N Engl J Med 377, 1919–1929, doi:10.1056/NEJMoa1709937 (2017). 28885881

[53] Socinski M. A. Atezolizumab for First-Line Treatment of Metastatic Nonsquamous NSCLC. N Engl J Med 378, 2288–2301, doi:10.1056/NEJMoa1716948 (2018). 29863955

[54] Gandhi L. Pembrolizumab plus Chemotherapy in Metastatic Non-Small-Cell Lung Cancer. N Engl J Med 378, 2078–2092, doi:10.1056/NEJMoa1801005 (2018). 29658856

[55] Wolchok J. D. Overall Survival with Combined Nivolumab and Ipilimumab in Advanced Melanoma. N Engl J Med 377, 1345–1356, doi:10.1056/NEJMoa1709684 (2017). 28889792 PMC5706778

[56] Peggs K. S., Quezada S. A., Korman A. J. & Allison J. P. Principles and use of anti-CTLA4 antibody in human cancer immunotherapy. Curr Opin Immunol 18, 206–213, doi:10.1016/j.coi.2006.01.011 (2006). 16464564

[57] Wolchok J. D. Long-Term Outcomes With Nivolumab Plus Ipilimumab or Nivolumab Alone Versus Ipilimumab in Patients With Advanced Melanoma. Journal of clinical oncology : official journal of the American Society of Clinical Oncology 40, 127–137, doi:10.1200/JCO.21.02229 (2022). 34818112 PMC8718224

[58] Palazon A. An HIF-1alpha/VEGF-A Axis in Cytotoxic T Cells Regulates Tumor Progression. Cancer Cell 32, 669–683 e665, doi:10.1016/j.ccell.2017.10.003 (2017). 29136509 PMC5691891

[59] Sharma P., Hu-Lieskovan S., Wargo J. A. & Ribas A. Primary, Adaptive, and Acquired Resistance to Cancer Immunotherapy. Cell 168, 707–723, doi:10.1016/j.cell.2017.01.017 (2017). 28187290 PMC5391692

[60] Lee J. H., Elly C., Park Y. & Liu Y. C. E3 Ubiquitin Ligase VHL Regulates Hypoxia-Inducible Factor-1alpha to Maintain Regulatory T Cell Stability and Suppressive Capacity. Immunity 42, 1062–1074, doi:10.1016/j.immuni.2015.05.016 (2015). 26084024 PMC4498255

[61] Doedens A. L. Hypoxia-inducible factors enhance the effector responses of CD8(+) T cells to persistent antigen. Nat Immunol 14, 1173–1182, doi:10.1038/ni.2714 (2013). 24076634 PMC3977965

[62] Bisilliat Donnet C. PHD2 Constrains Antitumor CD8+ T-cell Activity. Cancer immunology research 11, 339–350, doi:10.1158/2326-6066.Cir-22-0099 (2023). 36603132

[63] Gropper Y. Culturing CTLs under Hypoxic Conditions Enhances Their Cytolysis and Improves Their Anti-tumor Function. Cell Rep 20, 2547–2555, doi:10.1016/j.celrep.2017.08.071 (2017). 28903036

[64] Chang C. H. Metabolic Competition in the Tumor Microenvironment Is a Driver of Cancer Progression. Cell 162, 1229–1241, doi:10.1016/j.cell.2015.08.016 (2015). 26321679 PMC4864363

[65] Zappasodi R. CTLA-4 blockade drives loss of Treg stability in glycolysis-low tumours. Nature, doi:10.1038/s41586-021-03326-4 (2021). PMC8057670 33588426

[66] Li W., Cheng H., Li G. & Zhang L. Mitochondrial Damage and the Road to Exhaustion. Cell metabolism 32, 905–907, doi:10.1016/j.cmet.2020.11.004 (2020). 33264601

[67] Shi L. Z. Gfi1-Foxo1 axis controls the fidelity of effector gene expression and developmental maturation of thymocytes. Proc Natl Acad Sci U S A 114, E67–E74, doi:10.1073/pnas.1617669114 (2017). 27994150 PMC5224387

[68] Shen H. Selective suppression of melanoma lacking IFN-γ pathway by JAK inhibition depends on T cells and host TNF signaling. Nat Commun 13, 5013, doi:10.1038/s41467-022-32754-7 (2022). 36008408 PMC9411168

[69] Shen H. MicroRNA-30a attenuates mutant KRAS-driven colorectal tumorigenesis via direct suppression of ME1. Cell Death Differ 24, 1253–1262, doi:10.1038/cdd.2017.63 (2017). 28475173 PMC5520171

[70] Anders S., Pyl P. T. & Huber W. HTSeq--a Python framework to work with high-throughput sequencing data. Bioinformatics 31, 166–169, doi:10.1093/bioinformatics/btu638 (2015). 25260700 PMC4287950

[71] Love M. I., Huber W. & Anders S. Moderated estimation of fold change and dispersion for RNA-seq data with DESeq2. Genome Biol 15, 550, doi:10.1186/s13059-014-0550-8 (2014). 25516281 PMC4302049

[72] Kanehisa M., Furumichi M., Tanabe M., Sato Y. & Morishima K. KEGG: new perspectives on genomes, pathways, diseases and drugs. Nucleic Acids Res 45, D353–d361, doi:10.1093/nar/gkw1092 (2017). 27899662 PMC5210567

[73] Kuleshov M. V. Enrichr: a comprehensive gene set enrichment analysis web server 2016 update. Nucleic Acids Res 44, W90–97, doi:10.1093/nar/gkw377 (2016). 27141961 PMC4987924

