# Supplementary material for: HIF1α-glycolysis engages activation-induced cell death to drive IFN-γ induction in hypoxic T cells
Source: Res Sq. 2024 Jan 12:rs.3.rs-3830704. Preprint. [Version 1] doi: 10.21203/rs.3.rs-3830704/v1 (PMC10802708; doi:10.21203/rs.3.rs-3830704/v1)
Supplement: Supplement 1 [file NIHPPrs3830704v1-supplement-1.pdf]

Supplemental Figure 1

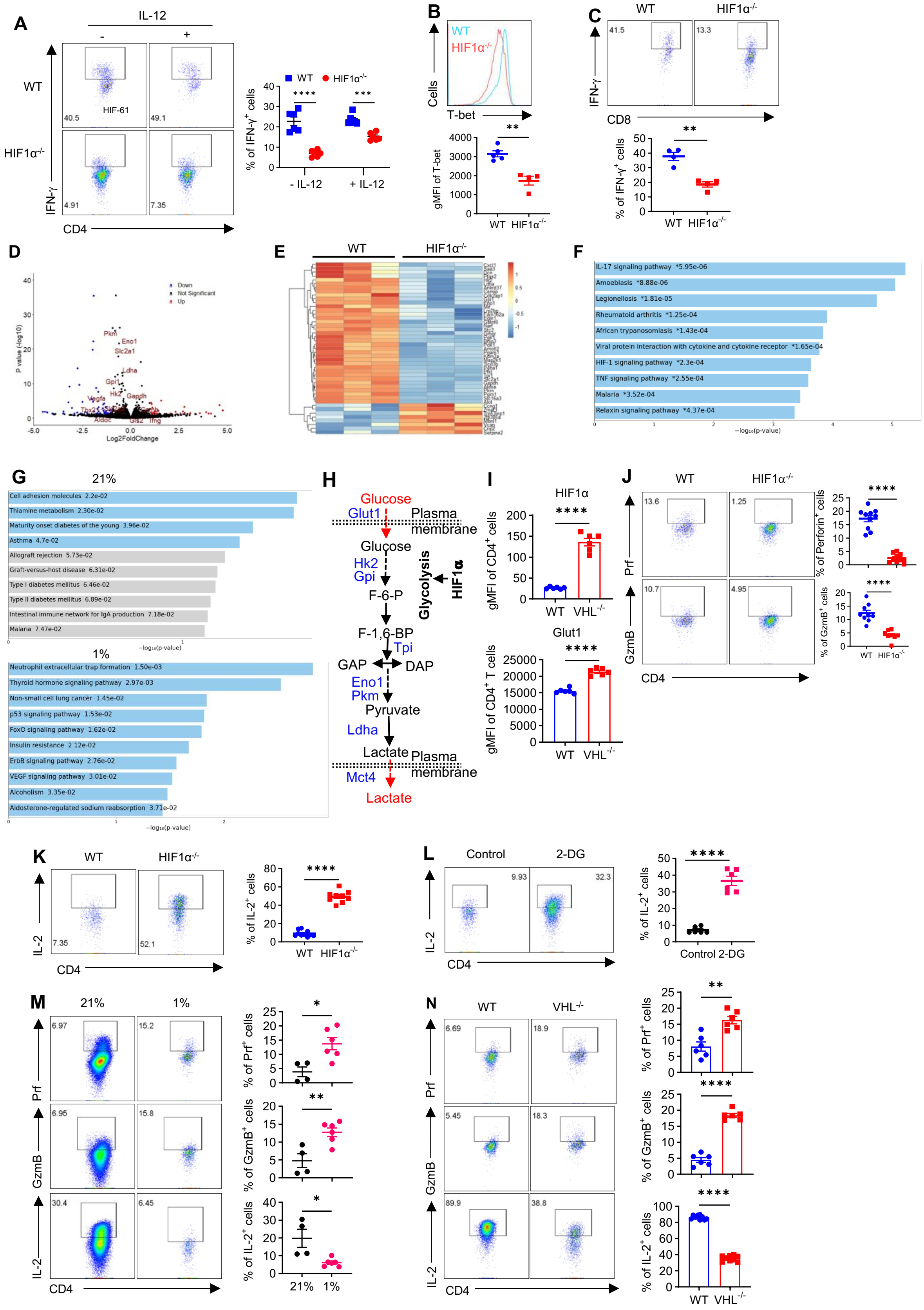

1039

Figure S1. HIF1α-glycolysis controls IFN-γ induction in hypoxic T cells, *in vitro*. **A**. Naïve CD4 T cells isolated from WT and HIF1$\alpha^{-/-}$ mice were activated under hypoxia for 5.5 days, with or without IL-12, followed by IFN-γ detection of . **B**. T-bet expression in WT and HIF1$\alpha^{-/-}$ CD4 T cells, activated under hypoxia for 2 days. **C**. Naïve CD8 T cells from WT and HIF1$\alpha^{-/-}$ mice were activated under hypoxia for 5.5 days and analyzed for IFN-γ production. **D-G**. Total RNAs extracted from WT and HIF1$\alpha^{-/-}$ CD4 T cells activated under normoxia for 48h were subjected to RNA-Seq. The gene expression analyses were performed using DESeq2 (version 1.34.0). The Wald test was used to calculate the p values and log2 fold changes. Genes with an adjusted p value <0.05 and absolute log2 fold change > 1 were considered as differentially expressed genes (DEGs). A volcano plot was used to show all upregulated and downregulated DEGs using the ggplot2 R package (**D**), with top 50 identified DEGs shown in a heatmap (**E**). Top 10 enriched signaling pathways (downregulated) from Enriched Kyoto Encyclopedia of Genes and Genomes (KEGG) analyses of DEGs were shown in **F**. Significant terms of the KEGG pathways were selected with a p value <0.05. **G**. Significantly upregulated top 10 enriched signaling pathways in HIF1$\alpha^{-/-}$ CD4 T cells activated under normoxia (21% O_2) (Upper Panel) and hypoxia (1% O_2) (Lower Panel). **H.** A diagram of the glycolytic pathway, with the major glycolytic genes highlighted in blue. **I**. Geometric mean fluorescence intensity (gMFI) of HIF1α (Top) and Glut1 (Bottom) in WT or VHL$^{-/-}$ CD4 T cells activated under hypoxia as in **A**. **J**. Production of perforin (Prf) and granzyme B (GzmB) by HIF1$\alpha^{-/-}$ CD4 T cells in hypoxia for 5.5 days. **K**. IL-2 production by activated WT and HIF1$\alpha^{-/-}$ CD4 T cells T cells activated in hypoxia for 5.5 days. **L**. IL-2 production by WT CD4 T cells activated under hypoxia for 5.5 days, treated with solvent (Control) or 0.5 µM of 2-DG. **M**. Production of Prf, GzmB, and IL-2 by CD4 T cells activated under normoxia (21% O_2) and hypoxia (1% O_2) for 5.5 days. **N**. Production of Prf, GzmB, and IL-2 by WT or VHL$^{-/-}$ CD4 T cells activated under hypoxia for 5.5 days. All the experiments were repeated at least twice. Pooled results shown in the dot plots and bar graphs depicted means ± SEM for all samples in each group, with each dot denoting an independent sample. *, p<0.05; **, p<0.01; ***, p<0.001; ****, p<0.0001. Source data were provided in the Source Data file.

Supplemental Figure 2.

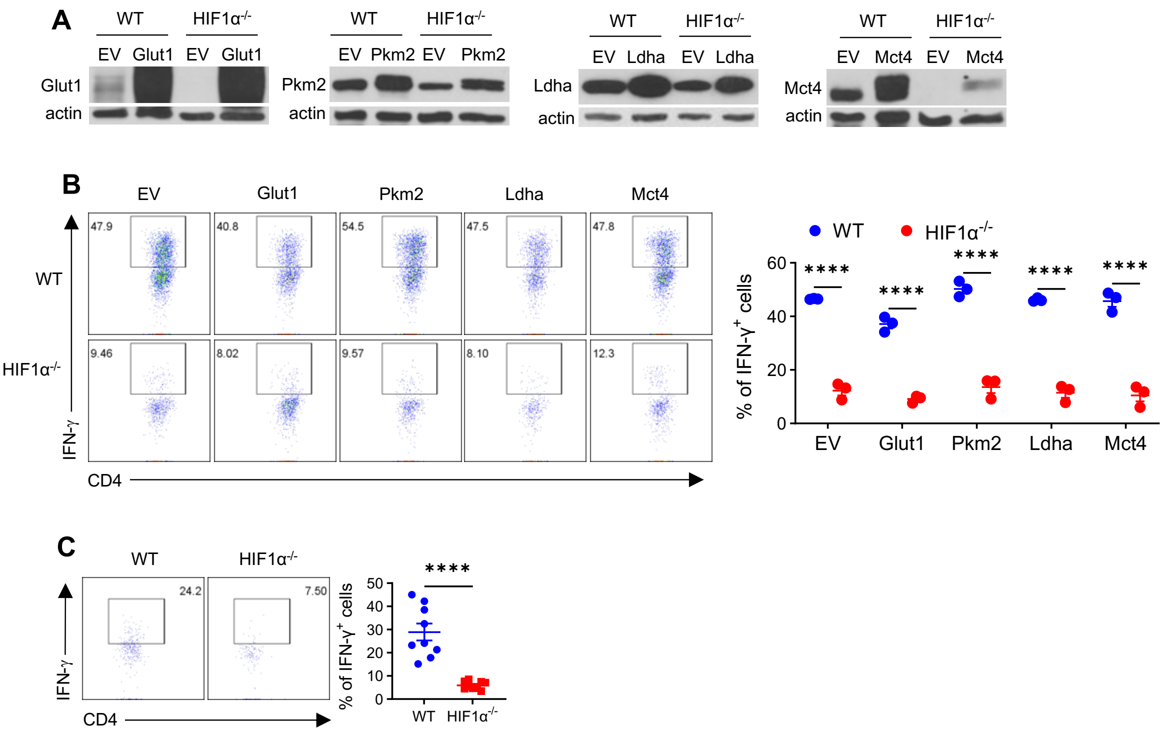

Figure S2. Direct regulation of IFN-γ induction in hypoxic T cells by HIF1α and intracellular acetyl CoA, *in vitro*. A. Protein expression of Glut1, Pkm2, Ldha and Mct4 in activated WT and HIF1α$^{-/-}$ CD4□ T cells successfully transduced (GFP□) with empty retroviruses (EV) or retroviruses expressing *Glut1*, *Pkm2*, *Ldha,* and *Mct4*, respectively. **B.** GFP□ T cells from **A** were activated under hypoxia for 5.5 days and analyzed for IFN-γ production. **C.** IFN-γ production by activated WT and HIF1α$^{-/-}$ CD4□ T cells co-cultured with MB49 cells for 48h, under hypoxia. All the experiments were repeated at least twice. Pooled results shown in the dot plots depicted means ± SEM for all the samples in each group, with each dot denoting an independent sample. ****, $p<0.0001$. Source data were provided in the Source Data file.

Supplemental Figure 3

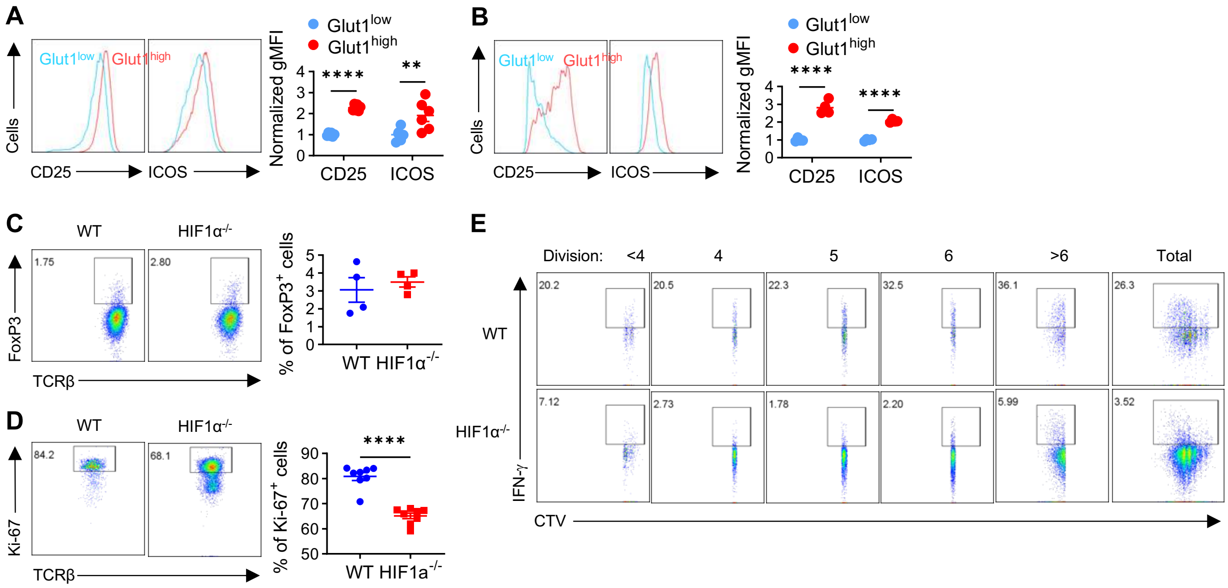

Figure S3. Reduced IFN-γ production by HIF1α$^{-/-}$ T cells is not due to proliferative defect. **A-B**. Naïve WT CD4□ (**A**) and CD8□ (**B**) T cells were activated under hypoxia for 2.5 days. Gated live Glut1^{low} and Glut1^{high} T cells were analyzed for the expression of ICOS and CD25, with geometric mean fluorescence intensity (gMFIs) shown in the dot scatter plot. **C-D**. Naïve WT and HIF1α$^{-/-}$ CD4□ T cells activated for 5.5 days under hypoxia were analyzed for FoxP3 (**C**) and Ki-67 (**D**). **E**. CTV-labeled naïve WT and HIF1α$^{-/-}$ CD4□ T cells were activated under hypoxia. CTV dilution and IFN-γ production were detected on Day 5.5 following activation. All the experiments were repeated at least twice. Pooled results shown in the dot plots depicted means ± SEM for all the samples in each group, with each dot denoting an independent sample. **, p<0.01; ****, p<0.0001. Source data were provided in the Source Data file.

Supplemental Figure 4

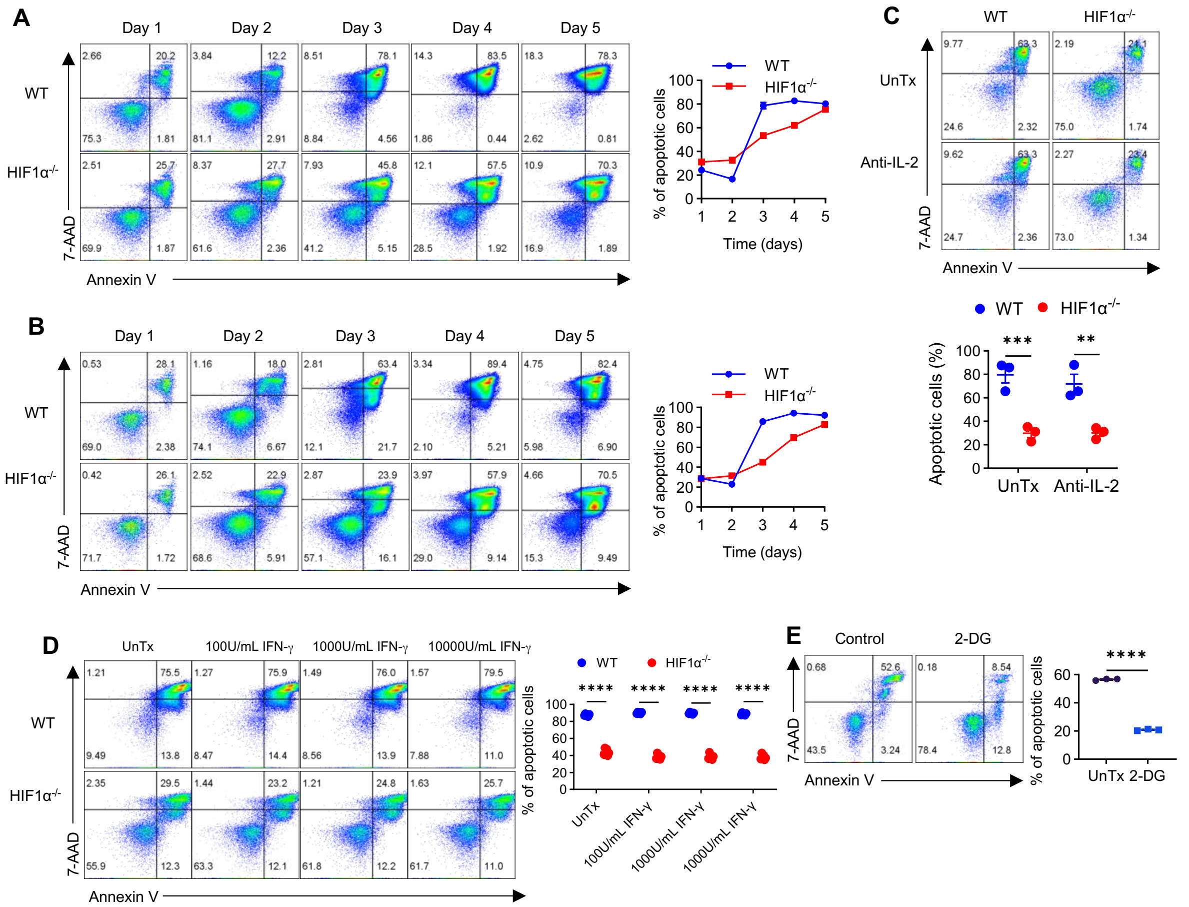

Figure S4. Attenuated AICD in HIF1α$^{-/-}$ T cells is unlikely mediated by extrinsic factors. A. Naïve WT CD4 T cells were activated under hypoxia for different times to assess cell death by staining for 7-AAD/Annexin V. The line graph on the right showed percentages of apoptotic cells. **B**. The time-course study as in **A** was repeated but with 50% of old media replaced by fresh media every day. **C-D**. Naïve WT and HIF1α$^{-/-}$ CD4 were activated under hypoxia for 3 days, with or without blocking antibodies against IL-2 (**C**), and with or without added IFN-γ (**D**), followed by analyses of cell death by 7-AAD/Annexin V staining. **E**. Naive human CD4 T cells isolated from PBMCs of healthy donors were activated under hypoxia for 3 days, in the absence and presence of 0.5mM 2-DG, followed by 7-AAD/Annexin V staining to detect cell death. All the experiments were repeated at least twice. Pooled results shown in the dot plots depicted means ± SEM for all the samples in each group, with each dot denoting an independent sample. **, p<0.01; ***, p<0.001; ****, p<0.0001. Source data were provided in the Source Data file.

Supplemental Figure 5

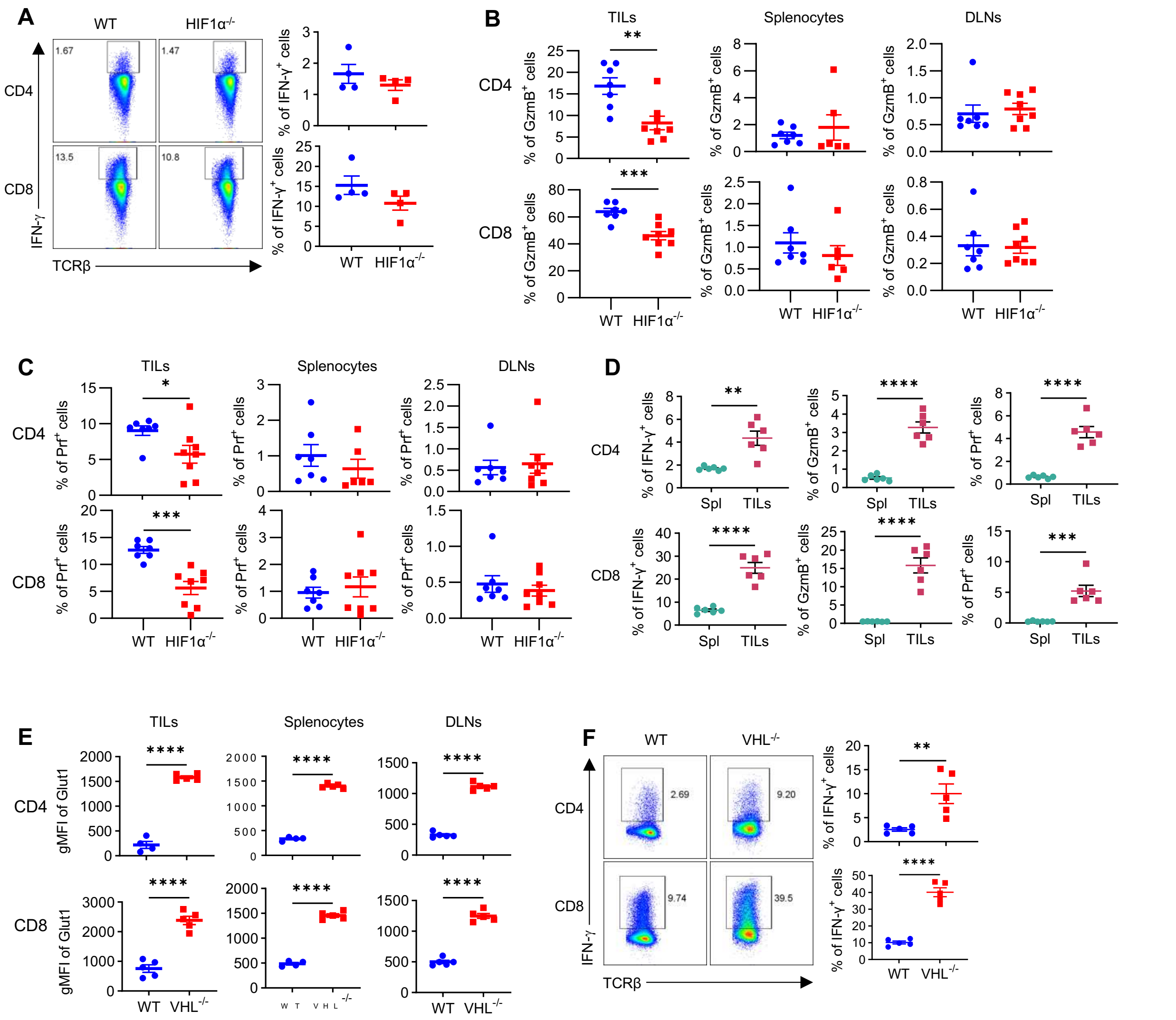

Figure S5. HIF1α controls effector function of TILs. A. T cells isolated from draining lymph nodes (DLNs) of WT or HIF1α$^{-/-}$ mice bearing established MB49 bladder tumor were analyzed for IFN-γ production. **B-C**. Production of GzmB (**B**) and Prf (**C**) by CD4^{+} and CD8^{+} TILs or CD4^{+} and CD8^{+} T cells from spleen and DLNs of WT or HIF1α$^{-/-}$ mice bearing established MB49 bladder tumor. **D**. Production of IFN-γ, GzmB, and Prf by CD4^{+} and CD8^{+} splenocytes vs CD4^{+} and CD8^{+} TILs, isolated from WT mice bearing MB49 bladder tumor. **E-F** T cells isolated from WT or VHL$^{-/-}$ mice bearing established MB49 bladder tumor were analyzed for Glut1 expression (**E**) in CD4^{+} and CD8^{+} TILs, splenocytes, and DLNs, as well as IFN-γ production by DLN CD4^{+} and CD8^{+} T cells. All the experiments were repeated 2-5 times. Pooled results shown in the dot plots depicted means ± SEM for all the mice in each group, with each dot denoting a mouse. *, p<0.05; **, p<0.01; ***, p<0.001; ****, p<0.0001. Source data were provided in the Source Data file.

Supplemental Figure 6

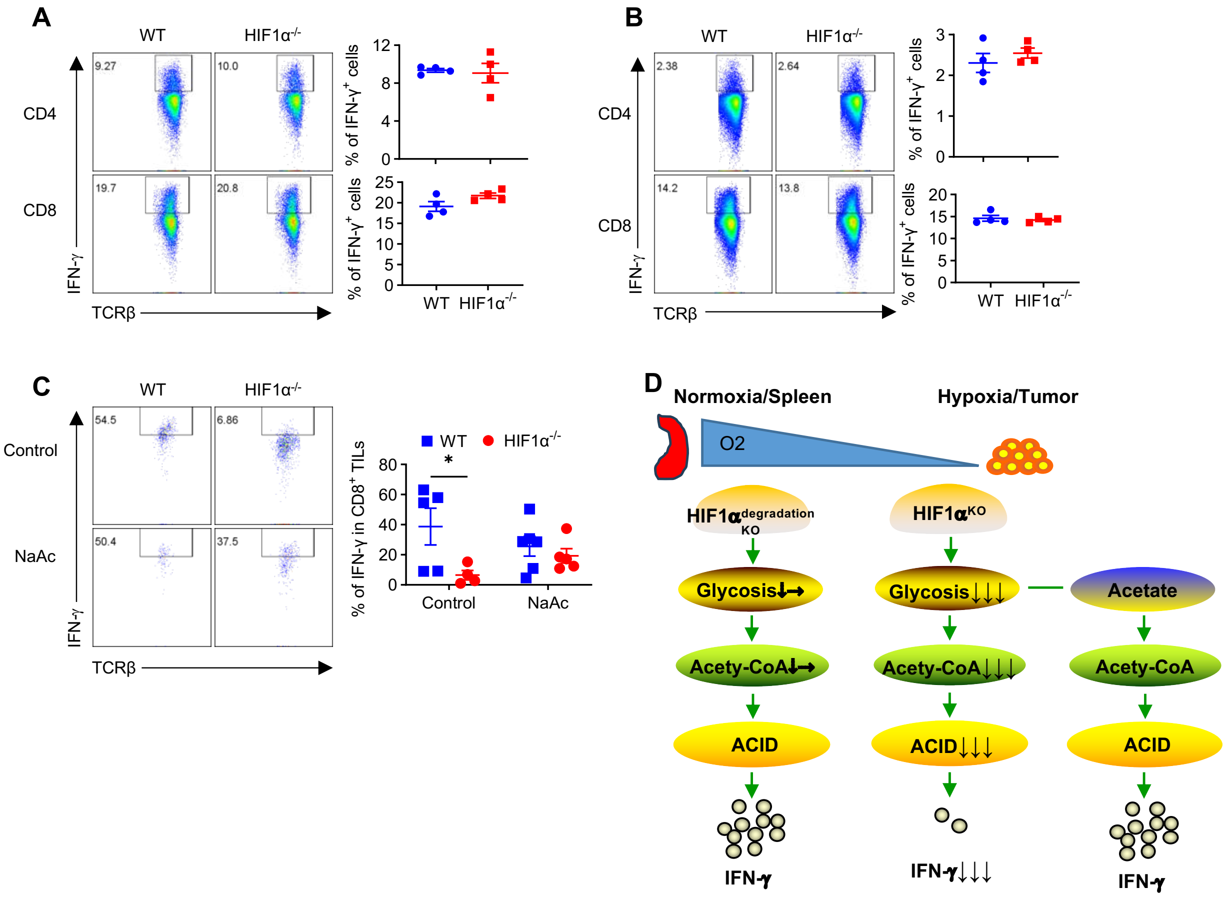

Figure S6. HIF1α in T cells governs therapeutic effects of ICBs. A-B. WT and HIF1α$^{-/-}$ mice bearing palpable MB49 bladder tumor were treated with combined anti-CTLA-4+anti-PD-1. IFN-γ production by CD4□ and CD8□ T cells from spleens (**A**) and draining lymph nodes (DLNs) (**B**) was shown. **C**. IFN-γ production by CD8□ TILs from MB49 bladder tumor-bearing WT and HIF1α$^{-/-}$ mice treated with combined anti-CTLA-4+anti-PD-1 alone or in conjunction with administration of sodium acetate (NaAc). All the experiments were repeated 2-5 times. Pooled results shown in the dot plots depicted means ± SEM for all the mice in each group, with each dot denoting a mouse. *, p<0.05; **, p<0.01; ****, p<0.0001. Source data were provided in the Source Data file. **D**. A proposed model of how T cell-intrinsic HIF1α-glycolysis controls IFN-γ production in hypoxic T cells.

Supplementary Figure 7. FACS sequential gating/sorting strategies

FACS strategy 1: for splenocytes, DLN, and cultured cells.

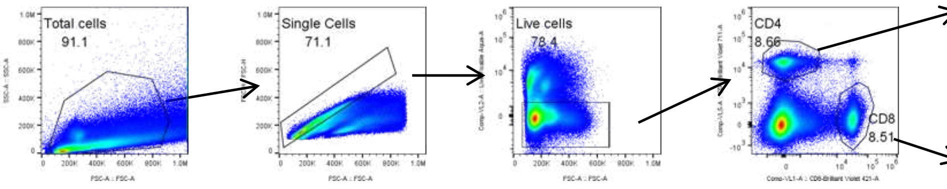

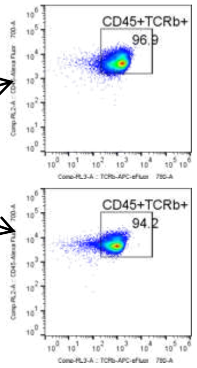

FACS strategy 2. for single cell suspensions of tumors.

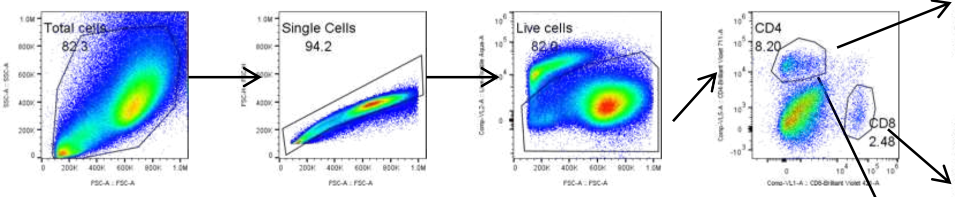

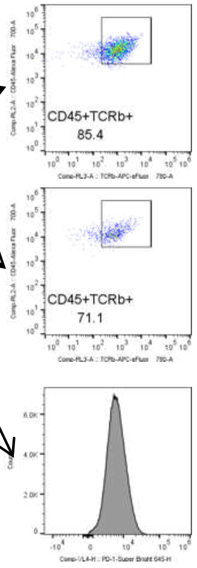

1191
1192

Supplementary Files

This is a list of supplementary files associated with this preprint. Click to download.

- SupplTable1HypoxiaWTvsHIF1aKO.xlsx
- SupplTable2NormoxiaWTvsHIF1aKO.xlsx
